# A large-scale genomic snapshot of *Klebsiella* spp. isolates in Northern Italy reveals limited transmission between clinical and non-clinical settings

**DOI:** 10.1038/s41564-022-01263-0

**Published:** 2022-11-21

**Authors:** Harry A. Thorpe, Ross Booton, Teemu Kallonen, Marjorie J. Gibbon, Natacha Couto, Virginie Passet, Sebastián López-Fernández, Carla Rodrigues, Louise Matthews, Sonia Mitchell, Richard Reeve, Sophia David, Cristina Merla, Marta Corbella, Carolina Ferrari, Francesco Comandatore, Piero Marone, Sylvain Brisse, Davide Sassera, Jukka Corander, Edward J. Feil

**Affiliations:** 1 Department of Biostatistics, University of Oslo, Oslo, Norway; 2 Bristol Veterinary School, University of Bristol, Bristol, UK; 3 Department of Clinical Microbiology, Turku University Hospital, Turku, Finland; 4 The Milner Centre for Evolution, Department of Life Sciences, University of Bath, Bath, UK; 5 Institut Pasteur, Université Paris Cité, Biodiversity and Epidemiology of Bacterial Pathogens, Paris, France; 6 Boyd Orr Centre for Population and Ecosystem Health, School of Biodiversity, One Health and Veterinary Medicine, College of Medical, Veterinary and Life Sciences, University of Glasgow, Glasgow, UK; 7 Parasites and Microbes, Wellcome Sanger Institute, Cambridge, UK; 8 Microbiology and Virology Unit, Fondazione Istituto di Ricovero e Cura a Carattere Scientifico Policlinico San Matteo, Pavia, Italy; 9 Romeo ed Enrica Invernizzi Pediatric Research Center, Department of Biomedical and Clinical Sciences Luigi Sacco, Università di Milano, Milan, Italy; 10 Department of Biology and Biotechnology, Università di Pavia, Pavia, Italy; 11 Department of Mathematics and Statistics, Helsinki Institute of Information Technology, University of Helsinki, Helsinki, Finland

**Keywords:** Bacterial genomics, Antimicrobial resistance

## Abstract

The *Klebsiella* group, found in humans, livestock, plants, soil, water and wild animals, is genetically and ecologically diverse. Many species are opportunistic pathogens and can harbour diverse classes of antimicrobial resistance genes. Healthcare-associated *Klebsiella pneumoniae* clones that are non-susceptible to carbapenems can spread rapidly, representing a high public health burden. Here we report an analysis of 3,482 genome sequences representing 15 *Klebsiella* species sampled over a 17-month period from a wide range of clinical, community, animal and environmental settings in and around the Italian city of Pavia. Northern Italy is a hotspot for hospital-acquired carbapenem non-susceptible *Klebsiella* and thus a pertinent setting to examine the overlap between isolates in clinical and non-clinical settings. We found no genotypic or phenotypic evidence for non-susceptibility to carbapenems outside the clinical environment. Although we noted occasional transmission between clinical and non-clinical settings, our data point to a limited role of animal and environmental reservoirs in the human acquisition of *Klebsiella* spp. We also provide a detailed genus-wide view of genomic diversity and population structure, including the identification of new groups.

## Abstract (web summary)

Genomic analyses of *Klebsiella* isolates sampled from multiple human, animal and environmental sources in Northern Italy explore *Klebsiella* population diversity and show that transmission of multidrug-resistant clones between clinical and environmental settings is scarce.

## Main

The *Klebsiella* genus is a member of the Enterobacteriaceae family. The most well studied species is *Klebsiella pneumoniae*, which the World Health Organization has recognized as a critical priority healthcare-associated pathogen^1^. Antibiotic resistance has spread rapidly within *K. pneumoniae* and other members of the genus since the early 1980s^2^; the plasmid-mediated spread of genes encoding carbapenemases over the last two decades is of particular concern^3,4^. Widespread clones of *K. pneumoniae* and other *Klebsiella* species associated with these genes are spread through the healthcare network^5^; in common with other key antimicrobial resistance (AMR) determinants, genes encoding carbapenemases have also been detected in multiple non-clinical settings including livestock and wastewater^6–8^. The potential public health risks posed by these non-clinical reservoirs of antibiotic resistance has led to a widening adoption of the One Health framework for AMR management^9^. This integrative approach is underpinned by a synthesis of antibiotic stewardship and AMR surveillance within clinical, community, agricultural and environmental settings. However, existing data on the abundance and distribution of AMR strains and genes in the environment does not provide a full picture and our current understanding of their maintenance and spread within and between ecological settings is fragmentary^10^. Given the urgent requirement for policy priorities informed by robust risk assessments, this represents a key knowledge gap.

A powerful way to infer pathogen transmission dynamics is to use whole-genome sequencing (WGS) combined with phylogenetic and other statistical analyses. WGS has been applied successfully within healthcare settings^11,12^ and played a key role in the management of the severe acute respiratory syndrome coronavirus 2 pandemic; however, capturing transmission pathways within complex non-clinical settings is more challenging. Large contemporaneous samples from well-defined regions are required to control for the confounding effects of divergence time and geographical variation. Nevertheless, a picture has begun to emerge that the risk of transmission of AMR genes and strains from environmental or agricultural settings into the clinic may be rather low, at least in well-resourced regions; this is a view seemingly at odds with the prevailing One Health cri de coeur^13^. Recent reports suggested that transmission of AMR strains and/or genes between humans and agricultural animals is limited in *Escherichia coli*^14^, *Enterococcus faecium*^15^ and *K. pneumoniae*^16,17^. The evidence, however, is equivocal^18^; a compelling counter-example is the study on colistin resistance dissemination in humans in China, which was largely driven by aquaculture activities^19^.

The sequencing of large and carefully sampled collections of isolates holds the promise to inform on the rate of transmission between settings^20^ and shed light on the relevant biological and ecological factors underpinning transmission barriers. While commonalities of gene and strain profiles between settings point to ample opportunities for mixing, the risks to public health of environmental reservoirs of AMR are difficult to gauge in the absence of this broad framework^10,21,22^. Recent advances in bioinformatics tools and analytical approaches provide the means to extract critical added value from genome data, enabling a more nuanced view of how microbes and mobile elements move through complex ecosystems.

In this study, we report a large-scale One Health study based on WGS data for 3,482 sequenced isolates, recovered from 6,548 samples. The sequenced genomes represent 15 *Klebsiella* species (including *Raoultella* species^23^) and approximately half (*n* = 1,705) are *K. pneumoniae*. These data informed a large-scale transmission analysis to identify environmental reservoirs likely to pose the greatest risk to public health. Samples from multiple clinical, community, veterinary, agricultural and environmental sources were taken within a 17-month period around a single city, Pavia, in Northern Italy. This represents an unprecedented contemporaneous sampling and sequencing effort within a restricted geographical area that is a known hotspot for healthcare-associated multidrug-resistant *K. pneumoniae*^5^. We describe the distribution of species, strains, AMR and virulence genes in different settings and provide detail on the phylogeny and diversity of the *Klebsiella* genus, including the identification of new lineages of potential species status.

## Results

### Sequencing, species assignments and phylogenetic analysis

**Fig. 1:**
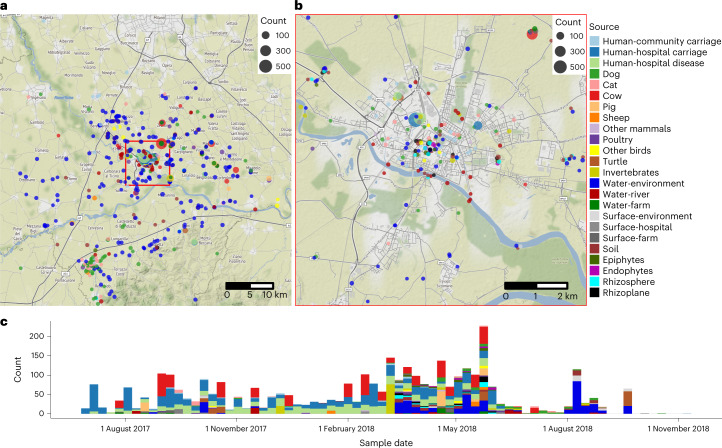
Summary of the sampling effort. **a**, Geographical summary of the whole sampling area. **b**, More detail of the region around the city of Pavia as highlighted by the red box in **a**. The size of each point indicates the number of samples and the colours represent the source. **c**, Timeline of the sampling effort broken down by source. Further details are hidden to preserve anonymity.

**Fig. 2:**
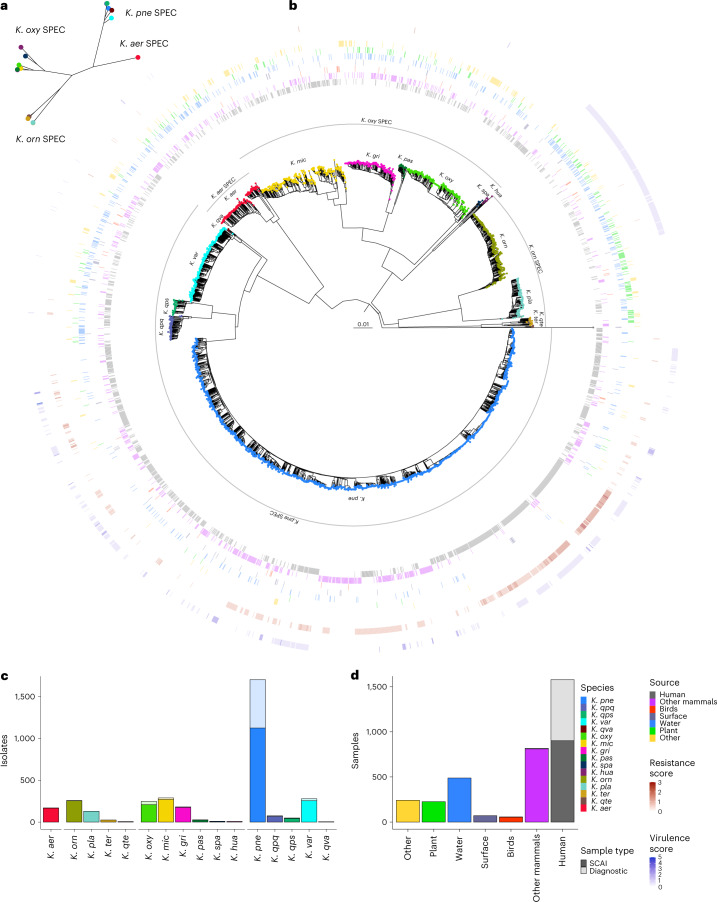
Phylogenetic tree with metadata and sample and source distributions. **a**, Maximum-likelihood phylogenetic tree constructed from core genes, coloured by species, with the SPECs shown. Only one isolate from each species is shown as this tree is intended to show the distances between species. **b**, Neighbour-joining phylogenetic tree constructed from pairwise Mash distances between all isolates, coloured by species, with the SPECs shown. The metadata rings show sources (inner rings) and resistance and virulence scores (outer rings). **c**, Bar plot showing the number of sequenced samples from each species. The dark bars show samples from SCAI media and the transparent ones show diagnostic samples. **d**, Bar plot showing the number of sequenced samples from each high-level source. The dark bars show samples from SCAI media and the transparent ones show diagnostic samples. With the following exceptions, the three-letter species abbreviations used are explained in the main text: *Klebsiella quasipneumoniae* subsp. *similipneumoniae* (*K. qps*); *Klebsiella planticola* (*K. pla*).

**Extended Data Fig. 1:**
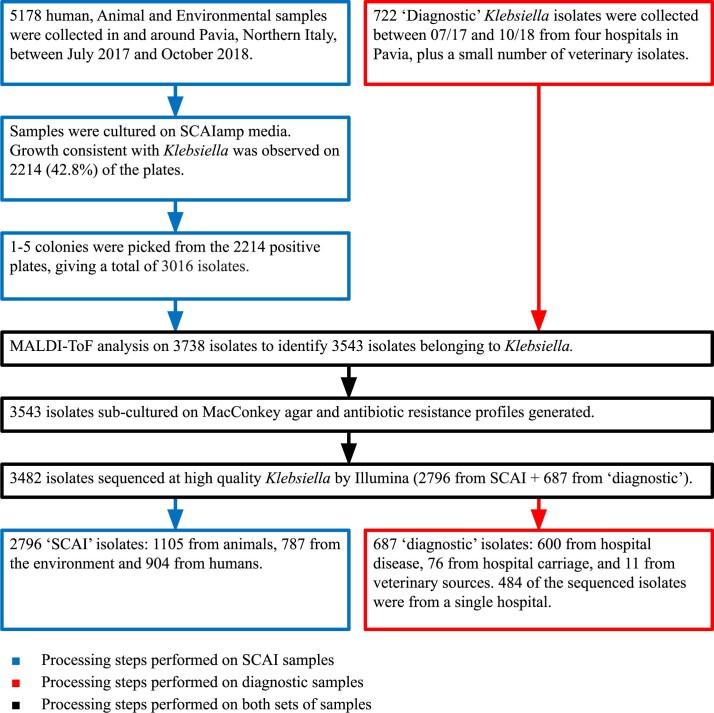
Summary of the sampling and subsequent methodology. Flowchart showing the number of initial samples taken, and the number of remaining samples after each processing step.

After quality control, 3,483 high-quality read sets and assemblies were retained: 2,796 from diverse sources recovered using Simmons Citrate Agar with Inositol (SCAI) media and 687 from ongoing clinical surveillance projects (summaries in Extended Data Fig. 1 and Supplementary Tables 1 and 2). Full isolate metadata, including species and lineage assignments, source, genotypic and phenotypic resistance data and phylogenetic trees are available for download from the Microreact project (https://microreact.org/project/KLEBPAVIA). A summary of the main metadata fields used in the Microreact project is provided in Supplementary Table 3. Figure 1 provides a summary of the sampling. A summary of the species assignments and sources of the 3,482 sequenced *Klebsiella* isolates and phylogenetic trees are given in Fig. 2. We used three-letter species abbreviations throughout this article; these are provided in the main text and the legend to Fig. 2.

**Extended Data Fig. 2:**
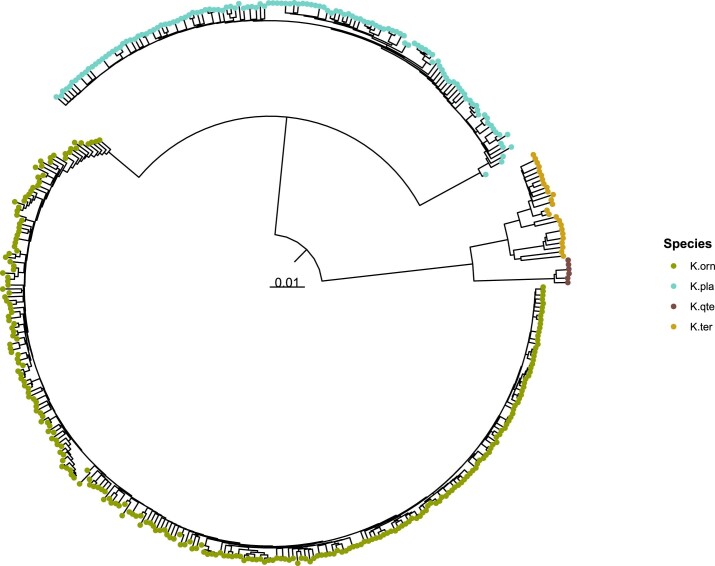
*K.orn* SPEC phylogeny. Circular phylogenetic tree for *K. ornithinolytica* and related species within the species complex *K.orn* SPEC. The tree was generated from Mash distances using the neighbour-joining method.

**Extended Data Fig. 3:**
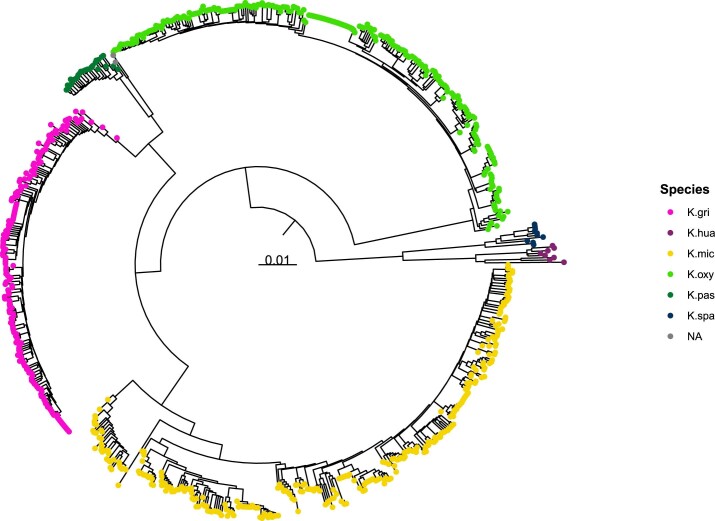
*K.oxy* SPEC phylogeny. Circular phylogenetic tree for *K. oxytoca* and related species within the species complex *K.oxy* SPEC. The tree was generated from Mash distances using the neighbour-joining method.

**Extended Data Fig. 4:**
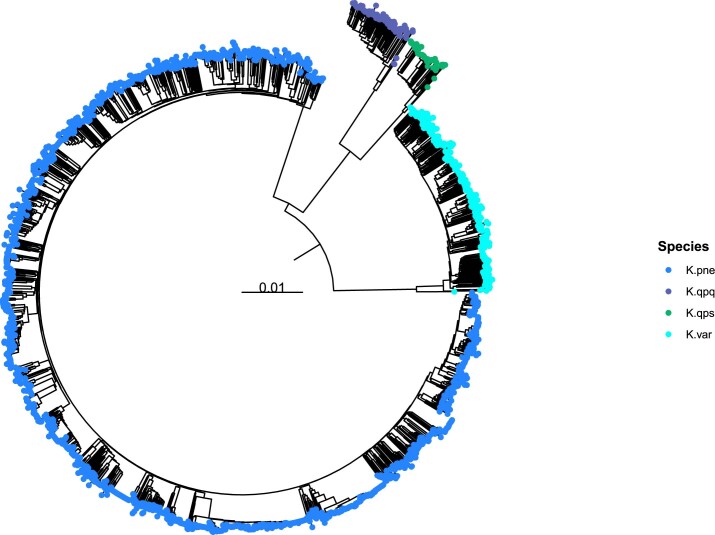
*K.pne* SPEC phylogeny. Circular phylogenetic tree for *K. pneumoniae* and related species within the species complex *K.pne* SPEC. The tree was generated from Mash distances using the neighbour-joining method.

We inferred a neighbour-joining tree of all 3,483 isolates using Mash^24^ distances (Fig. 2b) and generated a more statistically robust RAxML^25^ tree (GTR+Γ) based on a representative subset of 703 isolates (Fig. 2a). These trees were consistent with each other and with Kleborate^26^ species assignments, except for those cases where clusters were not present in the Kleborate database. We identified 15 recognized *Klebsiella* species, including *Klebsiella pasteurii* (*K. pas*) and *Klebsiella spallanzanii* (*K. spa*), which were first isolated during the course of this study^27^, and 8 isolates of *Klebsiella huaxiensis* (*K. hua*), which was previously only recovered from a urine sample in China^28^. Our data also resolved a new cluster of six isolates, to which we have assigned the label *Klebsiella quasiterrigena* (*K. qte*) (Extended Data Fig. 2) and two isolates from a hospital carriage that are positioned approximately equidistantly from *Klebsiella grimontii* (*K. gri*) and *K. pas* (labelled NA; Extended Data Fig. 3). A single isolate recovered from the surface of an automated teller machine (ATM) was assigned as a new species belonging to the genus *Superficiebacter*, designated *Superficiebacter* *maynardsmithii*^29^, and was retained as a convenient outgroup. Previous WGS studies did not support the assignment of the *Raoultella* species as a separate genus^7,23,30^, which is consistent with our data. Hence, we refer to these species as *Klebsiella*. Phylogenetic analyses revealed four higher-order clusters, which we have referred to as species complexes (SPECs), extending those used in^26^. These were named according to the canonical species in each group: *K. pneumoniae* (*K. pne* SPEC), *Klebsiella oxytoca* (*K. oxy* SPEC), *Klebsiella ornithicolytica* (*K. orn* SPEC (*Raoultella*)) and *Klebsiella aerogenes* (*K. aer* SPEC) (Extended Data Figs. 2–4).

*K. pneumoniae* (*K. pne*) was by far the most commonly sampled species, accounting for approximately half of the isolates (*n* = 1,705). This proportion was inflated by the inclusion of the 687 diagnostic isolates, 676 of which were sampled from healthcare settings. Of these isolates, 571 were *K. pne* (84%), confirming its dominance as a cause of human infection, with the opportunistic pathogen *K. oxy* being the second most common (*n* = 40; 6%) (Supplementary Table 4).

### Species clonality and population structures

**Fig. 3:**
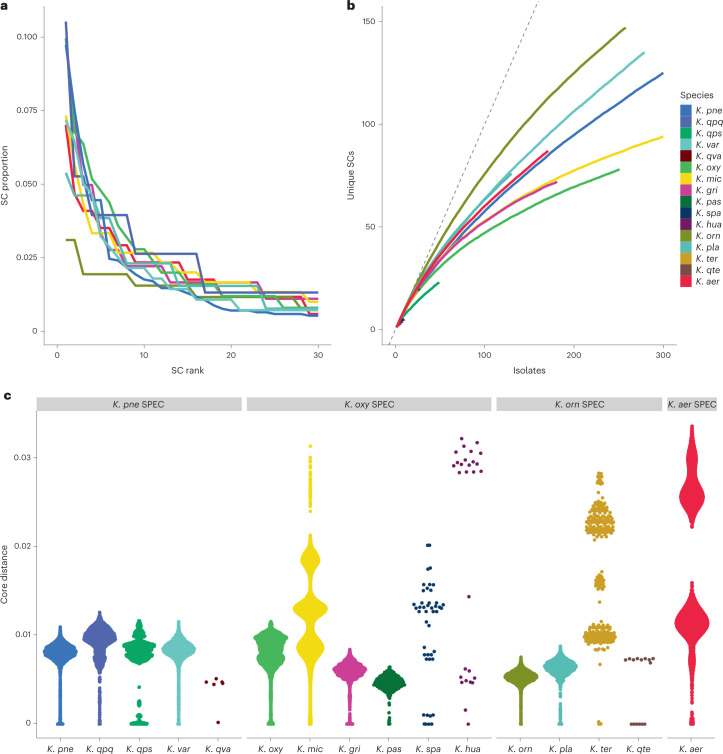
Clonality and population structure. **a**, Composition of the eight most common species as determined by SC frequencies. For each species, the isolates were grouped by SC and the SCs were ranked by their frequencies as a proportion of the dataset (top 30 SCs shown). **b**, The number of unique SCs as isolates were sampled. Accumulation curves were produced by randomizing the order of the isolates and counting the SCs, and then repeating this 100 times (mean values plotted). The dashed grey line indicates the *x* = *y* line. **c**, Distribution of pairwise core genome distances for each species. The distances were estimated using PopPunk and the points were arranged in the *x* direction by density to show their distributions.

**Extended Data Fig. 5:**
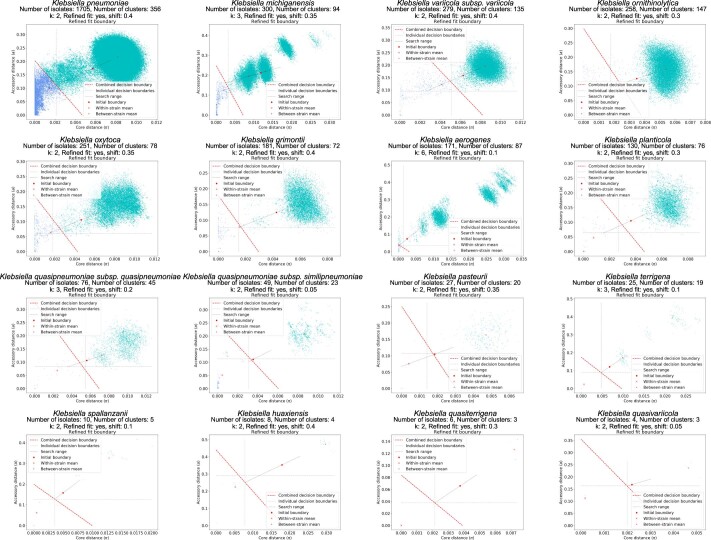
Lineage assignment with PopPunk. PopPunk was used to divide each species into sequence clusters (SCs). For each species, the number of components to fit in the mixture model (k) was chosen based on the scatter plot of core and accessory distances. The model was then fit, and the boundary refined using an iterative process of moving the boundary and reassessing the network features. In all cases the core boundary was used to define the clusters. The number of isolates, number of SCs, model K, and core distance boundaries are shown for each species.

**Extended Data Fig. 6:**
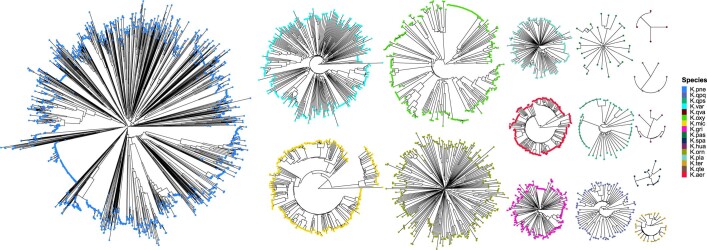
Phylogenies of individual species. Core genome phylogenetic trees for each species were generated using the neighbour-joining method.

We compared the population structures of the different species by delineating SCs using PopPunk^31^ (Extended Data Fig. 5 and Fig. 3). This revealed high levels of diversity in all species, as previously described for *K. pne*^32,33^. In total, we identified 1,168 SCs across all species, of which only 41 (3.5%) were represented by more than 10 isolates and 50% of all isolates corresponded to SCs that were observed no more than 6 times. The most common SC within each species represented between 3 and 10% of the population (Fig. 3a) and the SC accumulation curves were not close to saturation (Fig. 3b). *K. orn* showed particularly high diversity; 147 SCs were identified from 258 isolates and the most common SC accounted for 3% of the isolates. Pairwise divergences tended to be distributed around a modal average of approximately 1% (Fig. 3c) and each lineage was roughly equidistant to every other lineage (Extended Data Fig. 6). In some cases (for example, *K. pne*, *K. gri*), a much smaller peak was also evident at a much lower divergence, reflecting expansion of individual SCs. *Klebsiella michiganensis* (*K. mic*), *K. hua*, *K. spa*, *Klebsiella terrigena* (*K. ter*) and *K. aer* also showed more diverged modal peaks, with core genome distances up to 3%; this reflects the presence of deep subdivisions within these species, which is consistent with nascent speciation; this was also evident in the individual species trees (Extended Data Fig. 6 and Supplementary Table 5).

### Species are distributed non-randomly across different sources

**Fig. 4:**
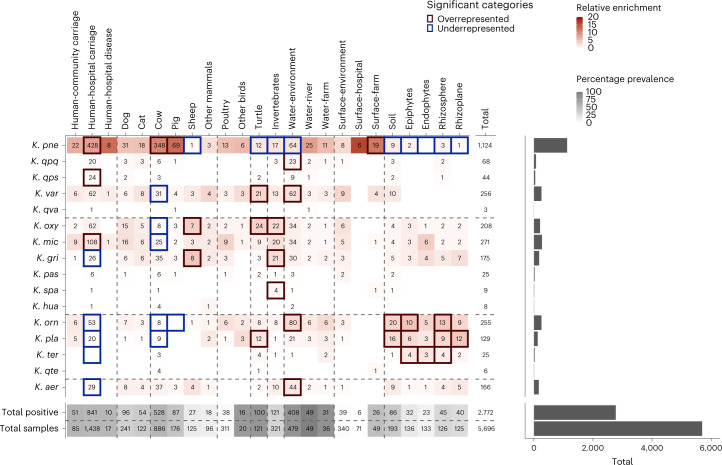
The distribution of species according to source. Only *Klebsiella* samples from the SCAI dataset (*n* = 2,795) are shown and 23 of these samples were removed either because they were from very poorly sampled sources (21) or could not be confidently assigned to a species (2). The rows represent species delimited according to SPECs and the columns represent sources delimited according to source categories. The grey shaded rows at the bottom of the table give the total number of positive samples for the corresponding source, and below, the total number of samples for that source. The grey shading reflects the percentage prevalence from each source. The number of positive samples are shown for each species from each source and a blank cell indicates zero positive samples. The red shading shows the relative enrichment of each species from each source, given the overall prevalence from that source and assuming a null hypothesis whereby all species would be equally likely to be observed from any given source. The dark red and blue borders show those categories where the number of samples is significantly higher or lower than expected, respectively, as determined by a permutation test. The bar plot to the right shows the number of samples from each species and the total sampling effort.

We explored the prevalence and distribution of the 15 recognized *Klebsiella* species and *K. qte* across different epidemiological and ecological sources (Figs. 2 and 4). Most of the disease isolates from hospital patients were recovered from diagnostic plates; thus, it was not valid to compare source prevalence between the diagnostic and SCAI samples. Therefore, the analysis presented in Fig. 4 was restricted to the 2,795 *Klebsiella* isolates recovered using the SCAI sampling strategy from any of the major source categories (*n* = 23 isolates excluded). Considering all sources, prevalence (calculated as the percentage of samples that were positive for at least 1 species) was highest for water samples (river, 100%; environmental, 85.2%; farm 86.1%) and turtles (82.6%), most of which are riverine. The source with the next highest prevalence was humans (hospital carriage, 58.5%; community carriage, 62.9%) and livestock (cows, 59.6%; pigs, 49.4%). The prevalence from soil was 44.6% and from plants 26.8%. While a high prevalence was observed from farm surfaces (53.1%), the prevalence from environmental and hospital surfaces was much lower (15.9%). Most species can be isolated from most sources; 20 sources harboured at least 7 species and 11 sources harboured at least 10 species.

We used a permutation test to gauge whether different species were non-randomly distributed between sources (Fig. 4). *K. pne* was significantly overrepresented in hospital carriage and in livestock (cows and pigs), as expected^16,34^, but was underrepresented in sheep, water (and turtles), invertebrates and soil/plants. Species within the *K. orn* SPEC were significantly overrepresented in soil and plants and underrepresented in hospital carriage, which is also consistent with previous work^34^. Other distributions were more surprising; for example, we did not find any evidence that *Klebsiella variicola* (*K. var*) is associated with plants, contrary to its original description^35^ and species from the *K. oxy* SPEC tended to be overrepresented in invertebrates. While this is consistent with a previous report of a symbiotic relationship between houseflies and *K. oxy*^36^, to our knowledge this specialism has not been described before in other species of this SPEC. These data also point to a significant overrepresentation of *K. mic* in hospital carriage and we note that a small but consequential proportion (17 out of 600; 2.8%) of the diagnostic isolates from hospital disease correspond to this species.

An important caveat with this analysis is that statistical association can result from clonality rather than ecological adaptation. For example, the apparent overrepresentation of *K. oxy* in turtles is due to the clonal expansion of a single lineage (*K. oxy* SC1) within a population of turtles in a pond at a botanical garden. However, we did not find evidence for clonal expansion of *K. mic* within hospital settings nor for certain *K. mic* lineages being more strongly associated with humans than others.

### Distribution of resistance genes

**Fig. 5:**
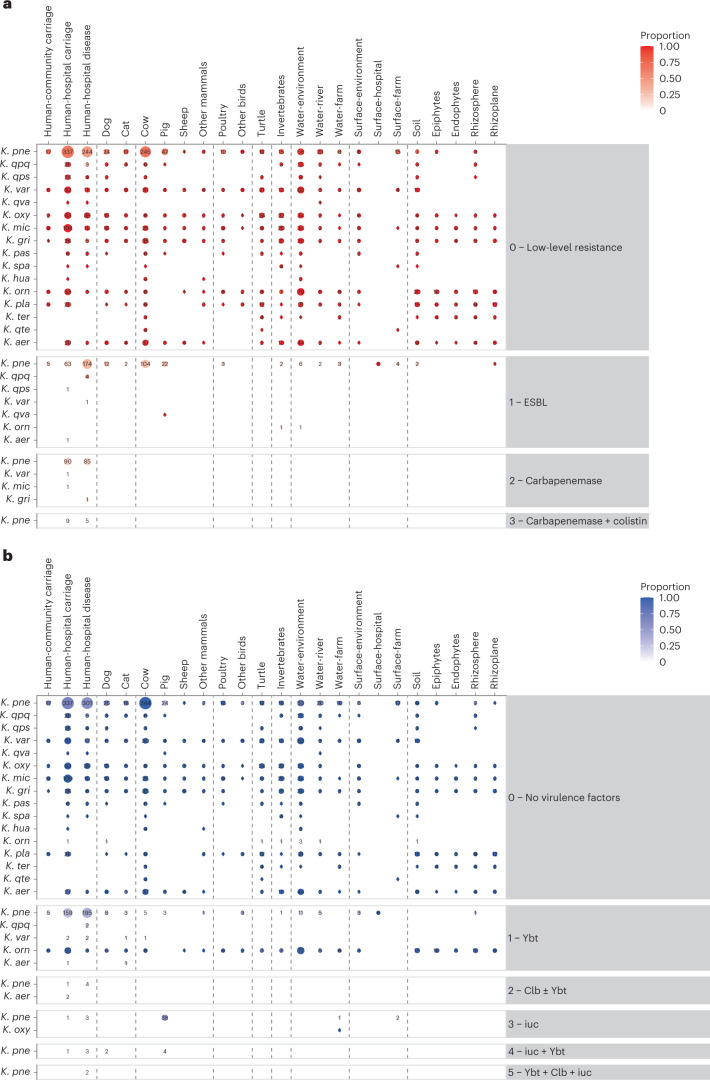
Distribution of resistance and virulence genes according to species and source. **a**, Resistance genes were identified and grouped into levels 0–3 by Kleborate. **b**, Virulence genes were identified and grouped into levels 0–5 by Kleborate. The area of the circles is proportional to the number of isolates and the text shows the number of isolates. The shading shows the proportion of isolates from a given species and source, which correspond to a given resistance or virulence level.

**Extended Data Fig. 7:**
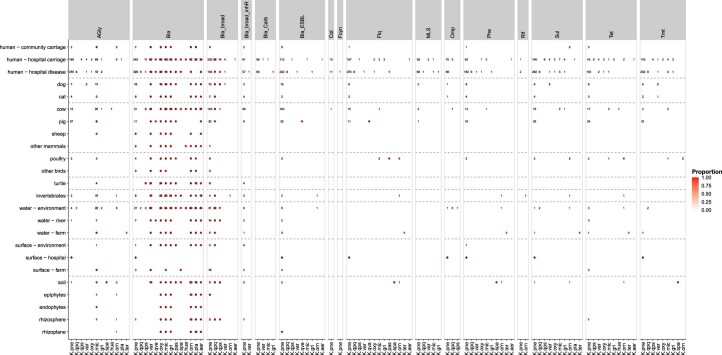
The distribution of resistance genes according to species and source. Detailed breakdown of the distribution of all resistance gene classes identified by Kleborate by species and source. The area of the circles is proportional to the number of isolates, and the text shows the number of isolates. The shading shows the proportion of isolates from a given species and source which harbour at least one gene from that class.

Kleborate^26^ assigns isolates to 1 of 4 resistance scores: 0 = low level resistance; 1 = extended-spectrum beta-lactamase (ESBL)-positive; 2 = carbapenemase-positive; and 3 = carbapenemase plus colistin-positive. The distribution of species according to these categories and to each source is shown in Figs. 2 and 5a; a full breakdown of resistance classes is shown in Extended Data Fig. 7; 82.4% (2,870 out of 3,482) of the isolates were category 0, with those scoring 1–3 being either *K. pne* from multiple sources or isolates of other species from hospital patients (exceptions are discussed below). None of the isolates, including *K. pne*, recovered from outside a hospital setting harboured a carbapenemase gene or showed phenotypic non-susceptibility to carbapenems.

Only three isolates of species other than *K. pne* from outside the hospital setting harboured an ESBL gene; in each case, the gene in question was *bla*_SHV-12_. These were a *K. orn* isolate recovered from a fly caught within a hospital (SPARK_2923_C1), a *K. orn* isolate from environmental water (SPARK_1613_C1) and a *Klebsiella quasivariicola* (*K. qva*) isolate from a pig (SPARK_1906_C1). Excluding *K. pne*, there were 9 isolates from other species recovered from hospital patients that harboured ESBLs (*bla*_CTX-M-15_, *n* = 4; *bla*_SHV-12_, *n* = 5). Of note are a pair of clonally related isolates (SPARK_1773_C1, SPARK_2031_C1) belonging to clone K.qpq_SC_11_ST571, which harboured *bla*_CTX-M-15_ plus the virulence factors *ybt*, *iro* and *rmpA*. These isolates were recovered from urine samples from two inpatients at the same hospital in April 2018. This is consistent with hospital transmission of a new *Klebsiella quasipneumoniae* subsp. *quasipneumoniae* (*K. qpq*) clone exhibiting both resistance and virulence genes.

**Extended Data Fig. 8:**
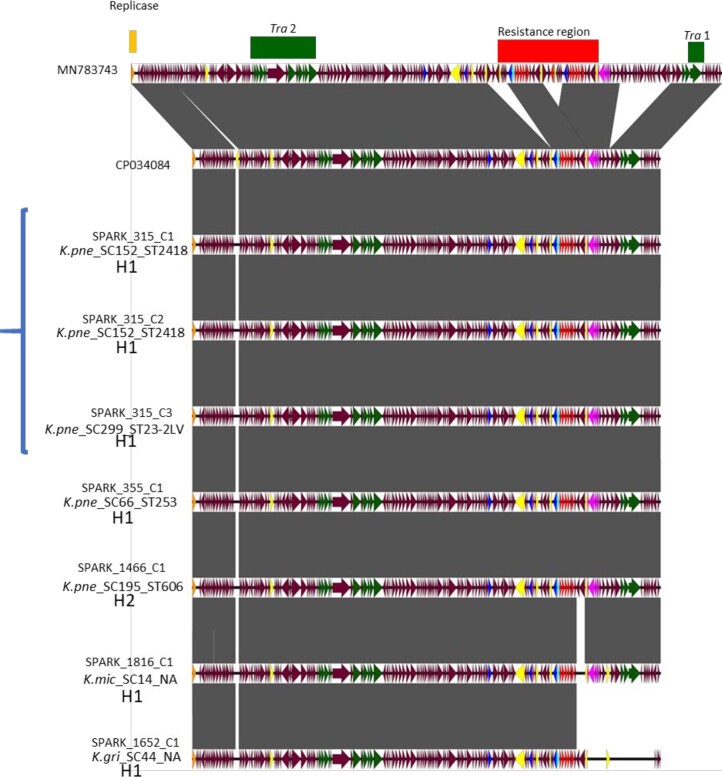
Alignment of *bla*_VIM_ plasmids. Alignment of the MN783743 and CP034084 reference plasmids with the contigs identified in our data as carrying the *bla*_VIM_ gene in IncA/C plasmids. Arrows represent the *bla*_VIM_ gene (turquoise), conjugal transfer system (green), resistance genes (red), mobile elements (yellow), hypothetical proteins (plum), replication protein *repA* (orange), integrases (dark blue) and the mercury resistance operon (pink). The three bracketed SPARK_315 SCAI carriage isolates were obtained from the same faecal sample from an outpatient in Hospital 1 (H1). The plasmid was found in two hospitals (H1 and H2). Two of these (SPARK_315_C1 and SPARK_315_C2) were members of the same clonal lineage (SC152_ST2418), whilst SPARK_315_C3 was an unrelated isolate related to the hypervirulent lineage ST23 (SC299_ST23-2LV). The plasmids are however essentially identical within all three isolates (which is identical to CP034084), indicating within-patient transfer. SPARK_355_C1 is a diagnostic carriage sample from a rectal swab from a different inpatient in Hospital 1 corresponding to lineage *K.pne*_SC66_ST253 and also contains the same VIM plasmid. SPARK_1466_C1 also contains a very similar plasmid. This isolate corresponds to a different lineage (*K.pne*_SC195_ST606) and is a diagnostic isolate from a urine sample from inpatient in Hospital 2. SPARK_1816_C1 is a *K.mic* diagnostic isolate (SC14) from a rectal swab from inpatient in Hospital 1 and contains also contains this plasmid. Finally, SPARK_1652_C1 is a *K.gri* diagnostic isolate (SC44), also from a urine sample from an outpatient in hospital 1. This isolate contains the same plasmid, but with a deletion near the resistance cassette.

Excluding *K. pne*, only three isolates from other species harboured a carbapenemase gene; these were all isolated from the hospital environment and carried *bla*_VIM-1_. Two of these (*K. mic* SPARK_1816_C1 and *K. gri* SPARK_1652_C1) presented nearly identical genotypic and phenotypic resistance profiles to each other and to five isolates of *K. pne*. This resistance profile is characterized by the presence of the *bla*_SHV-12_, *bla*_VIM-1_, *mph*(A) and *qnrS* genes, harboured by a class 1 integron (GenBank accession no. MN783743) associated with the conjugative IncA plasmid pR210-2-VIM^37^. This plasmid is known to circulate in multiple Enterobacteriaceae species in Italy^38^ and the re-emergence of VIM-1 in this region is thought to reflect the increased use of ceftazidime-avibactam against *K. pneumoniae* carbapenemase (KPC)-producing bacteria. Closer analysis revealed the presence of this plasmid in distinct *K. pne* clones within a single patient and in other *Klebsiella* species (Extended Data Fig. 8).

Regarding the 1,705 isolates of *K. pne*, 1,105 (64.8%) exhibited a low level of resistance (category 0), 411 (24.1%) carried an ESBL (category 1), 175 (10.3%) carried a carbapenemase gene (category 2) and only 14 (0.8%) carried a carbapenemase gene and colistin resistance (category 3). Two ESBL genes were dominant; *bla*_CTX-M-15_ and variants of *bla*_SHV-27_, which together accounted for 83.5% of all ESBL genes. These were distributed non-randomly between sources; 238 out of 256 (93%) of the *K. pne* isolates bearing *bla*_CTX-M-15_ were from humans, the exceptions being from hospital surfaces and companion animals. In contrast, only 51 out of 170 (30%) of the *K. pne* isolates bearing *bla*_SHV-27_ variants were from human sources compared to 87 out of 170 (51%) from cows. Of the 175 *K. pne* isolates harbouring carbapenemase genes, all were isolated from the hospital environment and the majority (*n* = 161; 92%) carried *bla*_KPC_ and corresponded to the healthcare-associated clones ST258/512 or ST307.

*K. pne*_ST307_SC1 was the most abundant clone in the dataset and was isolated from hospital surfaces and companion animals as well as hospital patients, although none of the ST307 isolates from non-human sources harboured *bla*_KPC_. Eleven *K. pne* isolates harboured *bla*_VIM-1_, including those discussed above, and 3 *K. pne* isolates harboured *bla*_OXA-48_. Of the 192 isolates with a carbapenemase gene for which phenotypic resistance data were also available, 91% showed phenotypic resistance to ertapenem, 71.7% to imipenem and 77.7% to meropenem. In contrast, the values were 0.8% (27 isolates), 0.18% (6 isolates) and 0.28% (9 isolates), respectively, for isolates (from all species) without a carbapenemase gene; these exceptions are likely due to changes in membrane permeability^39^. Consistent with the genotypic data, there was no evidence for any phenotypic resistance to carbapenems outside of the hospital environment.

There were 14 isolates in the highest resistance category; these were all *K. pne* isolates from hospitals and all harboured the carbapenemase gene *bla*_KPC_ plus a mutated *mgrB* gene known to confer colistin resistance. All except one of these isolates belong to the common healthcare-associated clone ST258/512, with the exception of a single ST307 isolate. Phylogenetic analysis of the 95 ST258/512 isolates suggested at least 5 acquisitions of the *mgrB* chromosomal mutation into this clone (Supplementary Fig. 1). The available phenotypic data confirmed resistance to colistin in 13 out of 14 of these isolates, 1 of which (SPARK_1222_C1) was originally assigned as sensitive using the BD Phoenix 100 automated system but was subsequently found to be resistant using the Sensititre platform. Phenotypic sensitivity to the other isolate containing *mgrB* (SPARK_372_C2) could not be confirmed because this isolate lost viability. In total, phenotypic resistance to colistin was observed in 46 *K. pne* isolates, 41 of which were from humans. Besides the 12 phenotypically resistant isolates harbouring an *mgrB* mutation, Kleborate did not detect a mechanism for colistin resistance in the other cases, including three *K. pne* isolates from pigs and a single *K. aer* isolate from a goat. This was not unexpected since many *mcr* variants are not included in the Kleborate database and colistin resistance can also be conferred through mutations responsible for membrane synthesis^40^. The final non-human colistin-resistant isolate was a single *K. pne* isolate from a cow that harboured *mcr-1*.

### Distribution of virulence genes

Like the genotypic resistance profiles, all isolates were assigned to 1 of 6 categories based on the presence of genes encoding the known virulence factors yersiniabactin (ybt), aerobactin (iuc), salmochelin (iro) and colibactin (clb), as identified by Kleborate (Figs. 2 and 5b); 2,749 out of 3,482 (78.9%) of all isolates and 1233 out of 1705 (72.3%) of *K. pne* isolates were in the lowest virulence category and 669 out of 3,483 (19.2%) of all isolates corresponded to virulence category 1, reflecting the presence of *ybt*, but the frequency of this locus varied markedly between species: *ybt* was present in 410 out of 1706 (24%) of the *K. pne* isolates, 249 out of 258 (96.5%) of the *K. orn* isolates, 6 out of 279 (2.1%) of the *K. var* isolates and 2 out of 171 (1.1%) of the *K. aer* isolates. The *ybt* locus in *K. orn* was assigned as an ‘unknown’ type by Kleborate, chromosomally located close to an transfer RNA-Asparagine site (with no evidence for an associated integrative conjugative element) and was phylogenetically distinct from the *ybt* locus in *K. pne*^41,42^. Despite being a core locus in *K. orn*, this distinct *ybt* variant was not found in any other species, including related species from *K. orn* SPEC.

**Extended Data Fig. 9:**
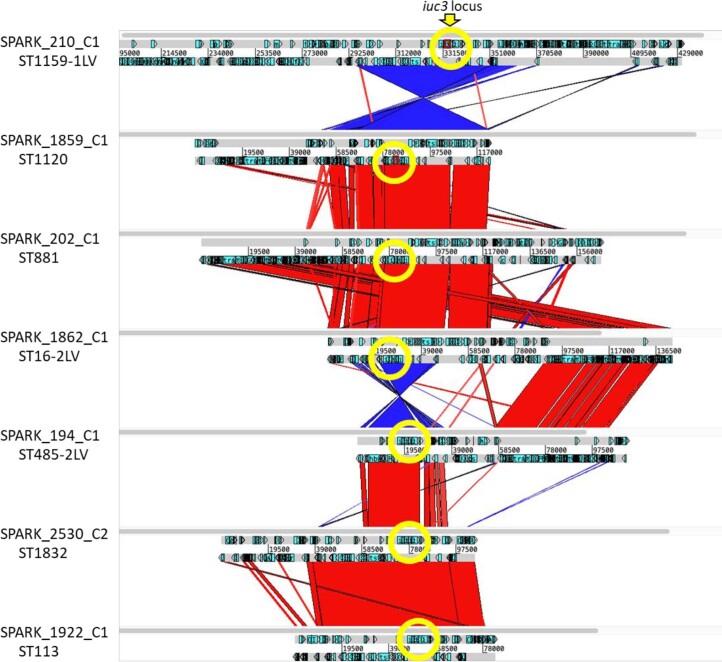
Comparison of *iuc3*-containing contigs from *K.pne*. Comparison of short-read contigs from *K.pne* isolates from pigs harbouring *iuc3* in this study. The ST and position of *iuc3* are shown. Contigs were compared with the Artemis Comparison Tool (ACT); sections of similarity of >500 bp are shown.

While only 7 isolates corresponded to virulence category 2 (*ybt* + *clb*), 45 *K. pne* isolates and 1 *K. oxy* isolate were assigned as virulence category 3. These isolates harboured the *iuc* locus that encodes the siderophore iuc and 38 out of 46 were recovered from pigs. In total, 42 out of 87 (48%) of the pig isolates harboured *iuc* and in 40 out of 42 (95%) cases harboured *iuc3*. Three *K. pne* isolates and one *K. oxy* isolate from the farm environment (water and surfaces) also harboured *iuc3;* a similar association between *iuc3* and pig isolates has recently been described in Germany^43^. The high frequency of *iuc3* in porcine isolates contrasts with clinical isolates, in which *iuc1* and *iuc2* are more common^44^. The porcine *iuc3* was observed on multiple sequence types (STs) and from different farms; hence, it is not a simple consequence of clonal spread. Preliminary analysis also suggests that *iuc3* is carried by diverse plasmids (Extended Data Fig. 9).

Twelve isolates were predicted to show a high level of virulence (categories 4 and 5). The two category 5 isolates corresponded to the hypervirulent lineage *K. pne* ST23 and contained all five virulence loci. These isolates were from patients in different hospitals and were sufficiently diverged to rule out epidemiological linkage. One of these ST23 isolates, SPARK_1158_C1, isolated from the urine of a hospital inpatient, had also acquired the resistance genes *qnrS1* and *bla*_TEM_ and exhibited phenotypic resistance to ciprofloxacin and levofloxacin. Of the ten *K. pne* isolates corresponding to category 4, four were from cases of hospital disease, four from pigs (all containing *iuc3*) and two from dogs. The two isolates from dogs, representing STs 5 and 25, harboured *ybt*, *iuc*, *iro* and *rmpA*.

### The distribution of sublineages below the species level

We examined the distribution across sources of subspecies SCs as defined using PopPunk, using the same permutation test used to examine species distributions (Supplementary Figs. 2–17). This analysis revealed that different *K. pne* lineages were associated with either cows or humans (Supplementary Fig. 2) and this was also borne out by phylogenetic analysis (Supplementary Fig. 18). The lineages SC1_ST307, SC2_ST17, SC3_ST512, SC4_ST45 and SC11_ST392 were mostly associated with humans, although these varied in the degree to which they were associated with hospital carriage versus hospital disease. For example, 66% of the SC1_ST307 isolates were associated with hospital disease and 28% with hospital carriage. These figures contrasted with K.pne_SC2_ST17, the second most common lineage in our dataset (*n* = 128), for which the equivalent figures were 20% and 57%, respectively. Other *K. pne* SCs were associated with cows rather than humans (for example, SC5_ST661, SC9_ST3068, SC10_ST2703, SC17_ST3345). Some intermingling occurred, particularly in SC5_ST661, which contained clonal expansions of both bovine and human isolates. This lineage was previously observed from both human and bovine sources^16^ and may represent a more generalist clone that is adapted to and able to transmit between both cows and humans.

Fewer statistically significant SC enrichments were apparent for other species, likely due to smaller sample sizes. However, a number of observations were notable. As discussed, *K. mic* was enriched within hospital carriage (Supplementary Table 4) but this was not due to the expansion of a single SC. Twenty-five of the 30 most common SCs of this species were present in hospital carriage samples but no single SC was significantly more commonly associated with hospital carriage relative to the others (Supplementary Fig. 3). In contrast, the association of *K. gri* with invertebrates was largely driven by *K. gri* SC1 (Supplementary Fig. 7). This is unlikely to reflect clonal expansion or sampling bias since *K. gri* SC1 was associated with different invertebrate hosts (a cockroach, fly, wasp and an unspecified bug) sampled in different locations. This clone, which has no notable resistance or virulence attributes, was also recovered from a cockroach caught in a hospital environment; an isolate very closely related to this clone was recovered from an outpatient of the same hospital (Supplementary Fig. 19).

### Quantifying transmission

**Fig. 6:**
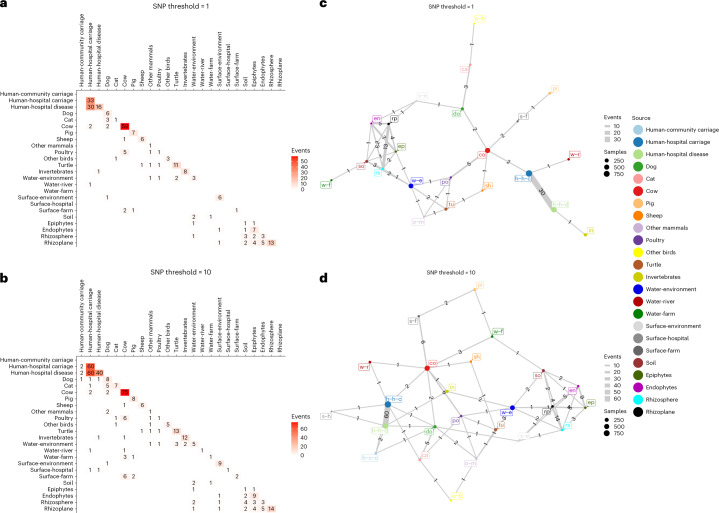
Transmission heatmaps and networks. **a**,**b**, Heatmaps showing the number of transmission events between each pair of sources, as determined by SNP thresholds of 1 (**a**) and 10 (**b**). The shading is proportional to the number of events and does not account for the number of samples from each source. **c**,**d**, Transmission networks showing the number of transmission events between each pair of sources, using the same data as in the heatmaps in **a** (**c**) and **b** (**d**), except that within-source events are not shown. The nodes represent the sources and the area of the node is proportional to the number of samples from that source. The edges show the number of transmission events and the thickness of the edge is proportional to the number of events between the two sources.

To quantify and compare transmission events between different settings, we used a single-nucleotide polymorphism (SNP) threshold-based approach (thresholds: 0, 1, 2, 5, 10, 20). It was clear from the resulting transmission matrices and networks (Fig. 6 and Supplementary Table 6) that most of the transmission occurred within a single source and, most importantly, that acquisition by humans almost always originated from other humans rather than from animals or the environment. In particular, our analysis further reinforces the view that transmission of *K. pne*, and other species, between cows and humans, which are the two most deeply sampled sources, is limited. Despite this, we note that sporadic transmission events occur relatively commonly between humans and companion animals and very occasionally between humans and other sources, including river water and invertebrates.

## Discussion

The One Health framework is integral to AMR research programmes aiming to mitigate the risks posed by non-clinical reservoirs of AMR through careful surveillance and stewardship. These risks exist on multiple levels, with the simplest being sporadic transmission events, for example, from livestock to farmers. If these events are epidemiologically distinct, and onward human-to-human transmission is unlikely, then the public health risk posed by each event is likely to be low. However, the recovery of isolates from dogs that are predicted to be hypervirulent, and the widespread healthcare-associated clone ST307 from both cats and dogs, suggest that transmission of high-risk clones between companion animals and humans may warrant close attention. We note that ST307 and ST15, the latter lineage being the most common *K. pne* lineage recovered from dogs in our data, have both previously been associated with companion animals^45^.

A more complex question relates to the emergence of high-risk lineages that combine heightened virulence and/or resistance attributes with the ability to move between, and spread within, different settings. A full understanding of the emergence of such clones requires a consideration of likely anthropogenic drivers, such as inappropriate use of antibiotics or other environmental stresses, but also of the underlying ecology since free transmission will be less likely if the selective landscape is characterized by local adaptation and specialization.

Our data revealed low levels of resistance and virulence genes outside of clinical settings and within species other than *K. pne*. Given the caveat that this may in part reflect biases in the database used by Kleborate towards well-characterized genes common in *K. pne*, this suggests that the emergence and subsequent spread of highly virulent and/or resistant lineages within the environment is a rare event. The acquisition of a variant *ybt* by *K. orn* as a core locus provides a possible exception, although the functional and epidemiological significance of this is unknown and it has not been observed in any other species. An additional example is the high frequency of porcine *K. pne* isolates that harbour the virulence locus *iuc3*. Although none of the pigs harbouring *iuc3* isolates showed signs of disease, it is likely that this *iuc* lineage is adaptive in the pig host. While much is unclear about the function and significance of the *iuc3* locus in *K. pne* isolates from pigs, and the near universal presence of *ybt* in *K. orn*, their close surveillance is warranted.

In contrast to the evidence from environmental and animal settings, our data provide evidence for the emergence of new and potentially high-risk lineages within the hospital setting, even in species other than *K. pne*. The new lineage *K. qpq* ST571, associated with hospital disease, is a worrying example since this clone harbours both resistance (*bla*_CTX-M-15_) and virulence (*ybt*, *iro* and *rmpA*) genes. Although only two isolates of this lineage were observed, they were both isolated from urine from different patients in the same hospital and are likely to be epidemiologically linked. A second example is the interspecies transfer of the plasmid pR210-2-VIM-like carrying *bla*_VIM-1_ from *K. pne* to *K. mic* and *K. gri* isolates, again within the hospital environment, as well as the intrapatient transfer of this plasmid between different *K. pne* lineages. Our data also revealed a surprisingly high rate of *K. mic* within hospital carriage, although in this case there was no evidence for the hospital spread of high-risk *K. mic* lineages. The high frequency of this species in hospital carriage, combined with the recovery of a *K. mic* strain harbouring the *bla*_VIM-1_, as well as previous reports of strains of this species harbouring carbapenemase genes^46^, urges heightened clinical awareness of this species.

We note that species and SCs within species are non-randomly distributed, with the clearest example being the distinct sets of *K. pne* SCs of human and bovine origin, which is consistent with previous studies^16^. Moreover, our analysis revealed that transmission is much more common within, than between settings, and that most cases of acquisition of *Klebsiella* by humans is likely to be from other humans, which has been suggested earlier for *E. coli*^47^. Thus, our data and analyses broadly challenge the view that bacterial strains and the resistance genes they carry can ‘flow’ unimpeded between different settings and we argue that local adaptation plays a role in mitigating transmission. The ecological and phylogenetic distribution of virulence and resistance genes also points to barriers to transmission between the clinical environment, animals and the environment. High levels of virulence and/or resistance tends to be rare in species other than in *K. pne* and outside the hospital environment. The complete absence of genotypic or phenotypic evidence for carbapenem non-susceptibility outside of healthcare settings is particularly noteworthy.

In conclusion, in this study we described WGS data incorporating multiple species of the *Klebsiella* genus from diverse sources. Our findings broadly corroborate recent research indicating hospitals as the hubs of *K. pne* resistance dissemination in Europe^5^ and justify a continued focus on breaking the transmission chains throughout the healthcare network. Moreover, our analysis suggested that new lineages of heightened virulence and/or resistance are most likely to emerge within hospital settings rather than in the environment or animals, although this possibility cannot be discounted. We contend that the One Health perspective is pertinent for restricting sporadic transmission events and that transmission dynamics will vary according to the region and the pathogen under study. For example, contact between humans and animals may be much more common in many low-resource regions^48^. We also recognize that the transmission dynamics of strains can be distinct from the plasmids they carry. Finally, we acknowledge limitations in our study with respect to wastewater and food-borne transmission, which may play important roles in the transmission cycle between humans, animals and the environment.

## Methods

### Sampling

A summary of the sampling and subsequent methodology is provided in Extended Data Fig. 1. In this study, two strategies were used to obtain samples: 5,900 samples were collected in the city of Pavia (Northern Italy) and the surrounding province between June 2017 and November 2018. Information on the samples collected is given in Supplementary Table 1. To summarize, the following types of samples were collected: stool and rectal swabs from hospital inpatients and outpatients (four different hospitals) and from a nursing home; stool from healthy volunteers; stool and rectal swabs from companion animals, farm animals and animals admitted to veterinary clinics (dogs, cats, cattle, pigs, poultry, turtles, rabbits and wild birds); invertebrates; samples of edible and ornamental plants, both wild and purchased from grocery shops, garden centres and wholesale distribution; soil samples; samples of drinking water (drinking fountains) and surface water (rivers and irrigation ditches); surface swabs from hospital, anthropic surfaces (including ATM keypads, ticket machines, buses, benches, supermarket trolleys) and farm surfaces (including enclosure, buckets and milking machines). Seven hundred and twenty-two *Klebsiella* isolates obtained from the laboratory diagnostic routine from urine, wound swabs, respiratory samples and blood cultures of hospital patients with infections were also processed. All sampling metadata was stored in Microsoft Excel 365 spreadsheets.

### Metadata

Detailed metadata on all the sequenced isolates is given in Supplementary Table 2 and the Microreact project. For guidance, a summary of the main metadata fields used in the Microreact project is provided in Supplementary Table 3 and instructions on the use of this platform are available at https://microreact.org/. Maps showing the locations of the sequenced isolates and a sampling timeline (sequenced isolates only) is given in Fig. 1.

### Sample processing

Stool and rectal swab samples (fecal swabs; Copan), both from human and animals, were enriched in Luria Bertani (LB) broth with amoxicillin (10 mg ml^−1^) at 36 ± 1 °C for 24 h. Invertebrates were frozen for at least 24 h after sampling and surviving bacteria were recovered from the surface of the animals as well as the gut. Freezing was carried out for practical and sanitary reasons, although this procedure was not optimal due to a loss of sensitivity and potentially some sampling bias resulting from bacterial cell death. For the surface, each insect was washed with sterile water for 2 min and an aliquot of the washing was enriched in LB broth with amoxicillin (10 mg ml^−1^). For the gut, the insect’s surface was washed with ethanol 70% for 5 min (once the surface had been sampled) and then air-dried. Small insects were ground with a pestle, while larger ones were dissected with a sterile scalpel to separate the gut. The gut was then used to inoculate LB broth with amoxicillin (10 mg ml^−1^), which was left to incubate at 36 ± 1 °C for 24 h.

For plants, each sample was divided into four portions: rhizosphere; rhizoplane; epiphyte; endophyte. The portions corresponding to the rhizosphere, rhizoplane and epiphytes were washed once with PBS (pH 7.2). The buffer used for washing was then added to the LB broth with amoxicillin (10 mg ml^−1^) at 36 ± 1 °C for 24 h. Endophytes were washed with ethanol 70% for 2 min (once the surface had been sampled) and rinsed with sterile water before being washed with a sodium hypochlorite 2% and Triton X-100 1% solution and incubated for 2 min before washing with sterile water. Endophytes were ground once in PBS (pH 7.2) with pestle and mortar. An aliquot of 1 ml was enriched in LB broth with amoxicillin (10 mg ml^−1^) at 36 ± 1 °C for 24 h.

Soil samples (5 g) were washed in PBS (pH 7.2), which was then added to the LB broth with amoxicillin (10 mg ml^−1^) at 36 ± 1 °C for 24 h. Water samples (1 l for both drinking and river water) were filtered through a sterile filter unit (pore size 0.2 µm; Thermo Fisher Scientific) and the membranes were enriched in LB broth with amoxicillin (10 mg ml^−1^) at 36 ± 1 °C for 24 h. For environmental water (mainly from ditches and ponds), we sampled and filtered at least 50 ml of water (higher volumes when possible) and then proceeded in the same way as the drinking and river waters. Surface swabbing was performed on areas of 10 cm^2^ for each point by using a swab rinse kit (Copan). After the collection, the swab and its medium were enriched in LB broth with amoxicillin (10 mg ml^−1^) at 36 ± 1 °C for 24 h. For each of the above samples, 1 μl of each enrichment was plated on SCAI^50,51^ medium and the plates were incubated at 36 ± 1 °C for 48 h.

### Diagnostic isolates

In addition to the *Klebsiella* isolates recovered using selective SCAI media from diverse sources, we also assembled an additional 722 diagnostic *Klebsiella* isolates recovered from hospital patients and veterinary clinics as part of ongoing surveillance programmes within the Pavia catchment area, of which 687 were successfully sequenced: 676 (98%) of the isolates were from human clinical cases, with 484 (70%) being from a single hospital, 600 (89%) were from hospital disease and 76 (11%) were from hospital carriage. Most of the human clinical isolates were recovered from urine (*n* = 417; 62%), the other sample types being blood (*n* = 74; 11%), rectal swab (*n* = 70; 10%), bronchial (*n* = 45; 7%), sputum (*n* = 20; 3%) and other minor sources.

### Species identification and antimicrobial susceptibility testing

Yellow and mucoid colonies on SCAI plates suspected to belong to the *Klebsiella* genus were identified at the species level through matrix-assisted laser desorption/ionization (MALDI)–time-of-flight (TOF) mass spectrometry (Microflex LT/SH; Bruker Daltonik GmbH) equipped with the Bruker biotyper 3.1 software. Once confirmed to be members of this genus, the isolates were subcultured on MacConkey agar for antibiotic susceptibility testing and DNA extraction. Antibiotic susceptibility was tested for all the isolates using the BD Phoenix 100 automated system and the dedicated panels NMIC-402 for all the diagnostic routine samples and NMIC-417 for all the other samples. The antibiotics and range of antibiotic concentrations present in the two panels are listed in Supplementary Table 7. We also used broth microdilution (Sensititre panel DKMGN; Thermo Fisher Diagnostics) on a subset of isolates to confirm phenotypic resistance to colistin in those cases where there were discrepancies between the genotypic and phenotypic data.

### DNA extraction, sequencing and bioinformatics

For all positive samples, genomic DNA was extracted from at least one colony using a QIAsymphony instrument (QIAGEN) and a dedicated kit (QIAsymphony DSP Virus/Pathogen; QIAGEN). All the extracts were stored at −80 °C. Genomic DNA libraries were prepared using the Nextera XT Library Prep Kit (Illumina) according to the manufacturer’s protocol. Illumina sequencing was performed at 3 centres: Wellcome Trust Sanger Institute, HiSeq X10, 150 base pair (bp), paired-end reads (*n* = 3,418); University of Bath, MiSeq, 250 bp paired-end reads (*n* = 110); MicrobesNG (Birmingham), HiSeq, 200 bp paired-end reads (*n* = 15), resulting in 3,543 isolates in total, of which 3,482 were confirmed as *Klebsiella* spp. and to be of high quality. The Illumina sequence reads were trimmed with Trimmomatic v.0.33 (ref. ^52^). SPAdes v.3.9.0 (ref. ^53^) was used to generate de novo assemblies from the trimmed sequence reads using *k*-mer sizes of 41, 49, 57, 65, 77, 85 and 93 and with the --cov-cutoff flag set to ‘auto’. The assemblies were annotated using Prokka v.1.12 (ref. ^54^). Kleborate v.2.0.0 (ref. ^26^) was used to group the isolates into *Klebsiella* species using Mash distances and assign multilocus sequence typing (MLST) types to several species. It was also used to detect antimicrobial resistance and virulence genes in the assemblies. Mob-Suite v.2.1.0 (ref. ^55^) and Prokka v.1.4.5 were also used to identify and annotate plasmids; Abricate v.1.0.0 (https://github.com/tseemann/abricate) was used to assign resistance genes to the plasmid contigs (PlasmidFinder and ResFinder databases downloaded on 21 May 2021 from https://bitbucket.org/genomicepidemiology/plasmidfinder_db and https://bitbucket.org/genomicepidemiology/resfinder_db, respectively). Two phylogenetic trees were generated to show species relationships across the whole genus. The first was a neighbour-joining tree of all isolates generated by RapidNJ from Mash v.2.0 (ref. ^24^) distances. The second was a maximum-likelihood tree of a subset of 703 isolates generated by RAxML v.8.2.8 (ref. ^25^) (GTR+Γ substitution model) from an alignment of core genes generated by Roary v.3.12.0 (ref. ^56^). Individual species trees were generated by PopPunk v.2.0.2 (ref. ^31^) core distances using the neighbour-joining method.

### MALDI–TOF

All of the sequenced isolates were initially assigned to 1 of 7 *Klebsiella* species by MALDI–TOF and, apart from 1 exception, subsequently assigned to 1 of 15 species on the basis of the genome sequences using Kleborate and phylogenetics. The accuracy of the MALDI–TOF assignments varied according to species; 88.4% of the isolates assigned as *K. pneumoniae* by MALDI–TOF were confirmed by WGS, whereas only 30% of the isolates assigned as *K. oxytoca* were confirmed as this species. These discrepancies reflect the fact that reference databases are currently unable to distinguish *K. oxytoca* from closely related species (Supplementary Table 8).

### Lineage assignment at the subspecies level

Kleborate was used to assign STs to the isolates but this was only possible for those species for which MLST schemes have been previously established. Therefore, we carried out intraspecies lineage assignments into SCs for all species using PopPunk v.2.0.2. For each species, the number of components to fit in the mixture model (*k*) was chosen based on the scatter plot of core and accessory distances. The model was then fitted and the boundary refined using an iterative process of moving the boundary and reassessing the network features. In all cases, the core boundary was used to define the clusters. For most species, 2 components provided the best fit, the exceptions being *K. aer* (*k* = 6), *K. mic*, *K. qpq* and *K. ter* (*k* = 3). For two species, there were outlying isolates (SPARK_1532_C1, SPARK_1532_C2; SPARK_1553_C1, SPARK_871_C1), which were removed to fit the model and then reassigned as query sequences. Plots of the final fits are shown in Extended Data Fig. 5. The SCs defined by PopPunk were named according to their relative abundance within each species, with SC1 being the most abundant, followed by SC2 and so on. For those species where STs could also be identified by Kleborate, the SCs defined by PopPunk matched closely with STs (Rand Index > 0.98), although SCs tended to be slightly more inclusive groupings. For ease of reference, we also used a compound identifier that combined SC with the corresponding canonical ST (for example, the most common group in *K. pne* was designated K.pne_SC1_ST307).

### Inferring transmission events

To quantify the frequency of transmission events within and between different sources, we aggregated data over all *Klebsiella* species and identified transmission events using a threshold-based approach based on SNP distances using a mapping procedure. Briefly, for each SC in the dataset we used the ‘sample_n’ function from the dplyr v.0.8.3 package in R v.3.6.1 to randomly choose a reference isolate to represent the SC. This was an objective and practical solution, given the high diversity of the dataset (1,168 SCs across 15 species). We mapped all isolates from a given SC to this random SC-specific reference using Snippy v.4.6.0 (https://github.com/tseemann/snippy), with variant calling by freebayes v.1.3.2 and the parameters --mapqual 60, --basequal 13, --mincov 10, --minfrac AUTO, --minqual 100, --maxsoft 10. We then used snp-dists v.0.7.0 (https://github.com/tseemann/snp-dists) to count the number of SNPs between each pair of isolates from that SC.

**Extended Data Fig. 10:**
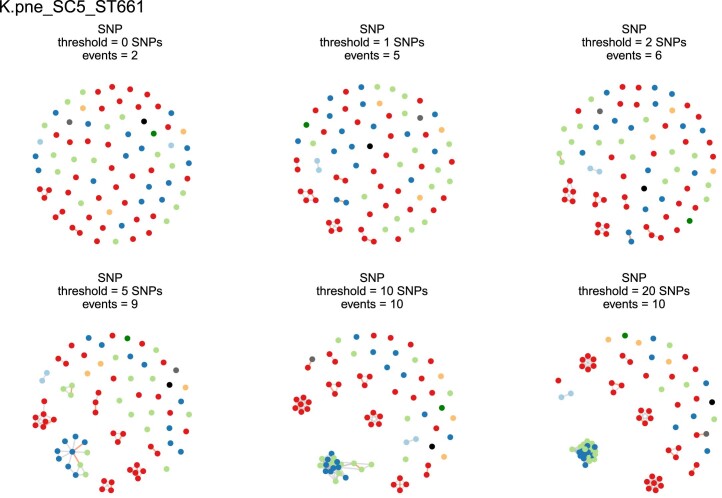
Transmission clusters. Networks were used to identify transmission clusters using multiple SNP thresholds. To ensure that the estimates were conservative, only one event was counted for each pair of sources that satisfied the threshold in each component of the network. These events are denoted by the red edges. Only one SC is shown as an example of the method.

For each SC, we created a network where all isolates were connected to all other isolates and then pruned this network into ‘putative transmission clusters’ by removing all the links between isolates where the SNP distance was greater than the chosen threshold SNP distance (an example is given in Extended Data Fig. 10). For each of these putative transmission clusters, we recorded every unique source pair (including same source pairs) connected by a pair of isolates differing by an SNP distance ≤ threshold. We excluded pairs of samples from the same individual hosts. Each unique source pair (not isolate pair) within each cluster, which satisfied these criteria, was recorded as a single transmission event. This approach is conservative because multiple transmission events occurring within a cluster between any given source pair are only counted once, but this avoids double counting of single transmission events. To obtain transmission frequencies, we normalized the count of transmission events for each source pair by the total number of isolates in each pair of sources. We carried out this analysis using 6 different SNP thresholds: 0, 1, 2, 5, 10 and 20 SNPs. The upper threshold (20 SNPs) was informed by a recent analysis of *K. pne* transmission within and between healthcare settings^5^.

### Statistics and plotting

All statistics and plotting were performed with R v.3.6.1. The permutation tests were done by randomly permuting the source categories and cluster labels (either species or SC depending on the dataset) 10,000 times and then deriving a *P* value by dividing the number of permutations that were at least as extreme as the observed data by 10,000. The test was two-tailed; therefore, a significance level of 0.025 was used at each tail. The *P* values were corrected using the Benjamini–Hochberg method from the p.adjust function in R. Other tests of association were done using the chi-squared test. All the plotting was done in ggplot2 v.3.3.5 and the multi-panel plots were produced using cowplot v.1.1.0.

### Ethical approval

This study was approved by the ethical committee of the San Matteo Hospital in Pavia under no. 20170001787 on 25 May 2017. The proceeding number is 2017000759 and the internal code of the project is 0890170117. The ethical procedure includes written informed consent from all the patients participating in the study.

### Reporting summary

Further information on research design is available in the Nature Portfolio Reporting Summary linked to this article.

### Supplementary information

Supplementary Information Supplementary Figs. 1–19.

Reporting Summary

Supplementary Tables Supplementary Table 1: Metadata summary of all samples collected. Supplementary Table 2: Metadata of all sequenced isolates. Supplementary Table 3: Explanation of metadata fields. Supplementary Table 4: Summary of the species distributions of 1,540 hospital-derived isolates in the SCAI (S) and Diagnostic (D) strain collections. Most of the carriage isolates are from the SCAI collection and most of the disease isolates are from the diagnostic sample. Table S5: Individual species trees in Newick format. Supplementary Table 6: Transmission matrices for all SNP thresholds. Supplementary Table 7: List of antibiotics and range of concentrations tested in the BD Phoenix NMIC-402 and BD Phoenix NMIC-417 panels. Supplementary Table 8: Comparison of species assignments by MALDI–TOF and whole-genome sequencing. Supplementary Table 9: Accession numbers for all sequenced isolates in the study.

## Data Availability

All raw Illumina sequence data are available from the European Nucleotide Archive under accession no. PRJEB27342. The individual accession numbers for all sequenced isolates are in Supplementary Table 9. The metadata and phylogenetic analysis are available for download from the Microreact project https://microreact.org/project/KLEBPAVIA; additional metadata is provided in Supplementary Tables 1 and 2. The PlasmidFinder and ResFinder databases were downloaded on 21 May 2021 from https://bitbucket.org/genomicepidemiology/plasmidfinder_db and https://bitbucket.org/genomicepidemiology/resfinder_db, respectively. Map tiles by Stamen Design, under CC BY 3.0. Data by OpenStreetMap, under ODbL.

## Code availability

The R code for the transmission analysis and permutation tests is available from https://github.com/harry-thorpe/SPARK_code.
