# Supplementary material for: A large-scale genomic snapshot of Klebsiella spp. isolates in Northern Italy reveals limited transmission between clinical and non-clinical settings
Source: Nat Microbiol. 2022 Nov 21;7(12):2054–67. doi: 10.1038/s41564-022-01263-0 (PMC9712112; doi:10.1038/s41564-022-01263-0)
Supplement: Supplementary file 1 — Supplementary Figs. 1–19. [file 41564_2022_1263_MOESM1_ESM.pdf]

nature microbiology

Article https://doi.org/10.1038/s41564-022-01263-0

# A large-scale genomic snapshot of *Klebsiella* spp. isolates in Northern Italy reveals limited transmission between clinical and non-clinical settings

In the format provided by the authors and unedited

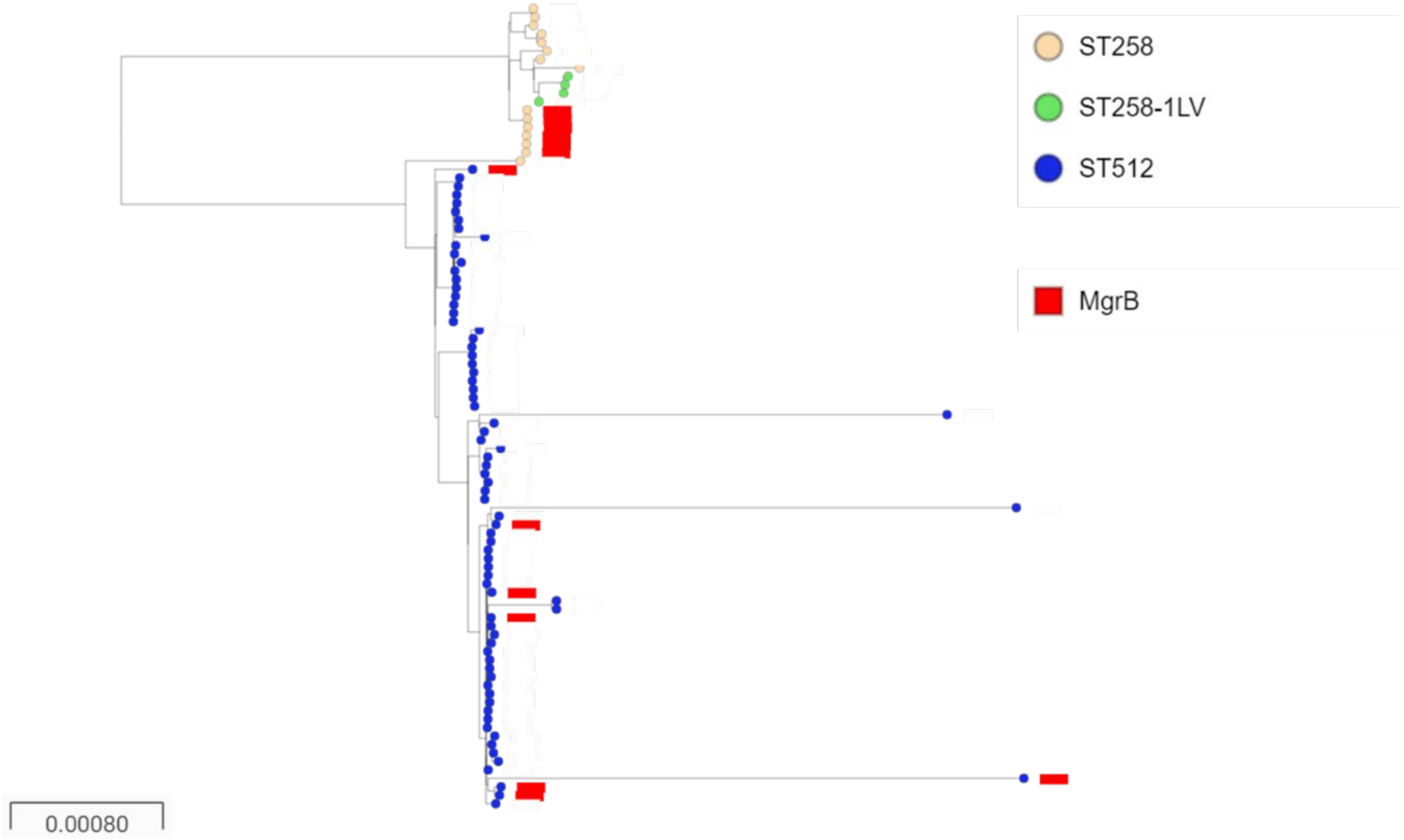


**Figure S1. Phylogenetic analysis of *K.pne* ST258/512.** The presence of the mutation in *mgr*B conferring colistin resistance in *K. pne* clone ST258/512.

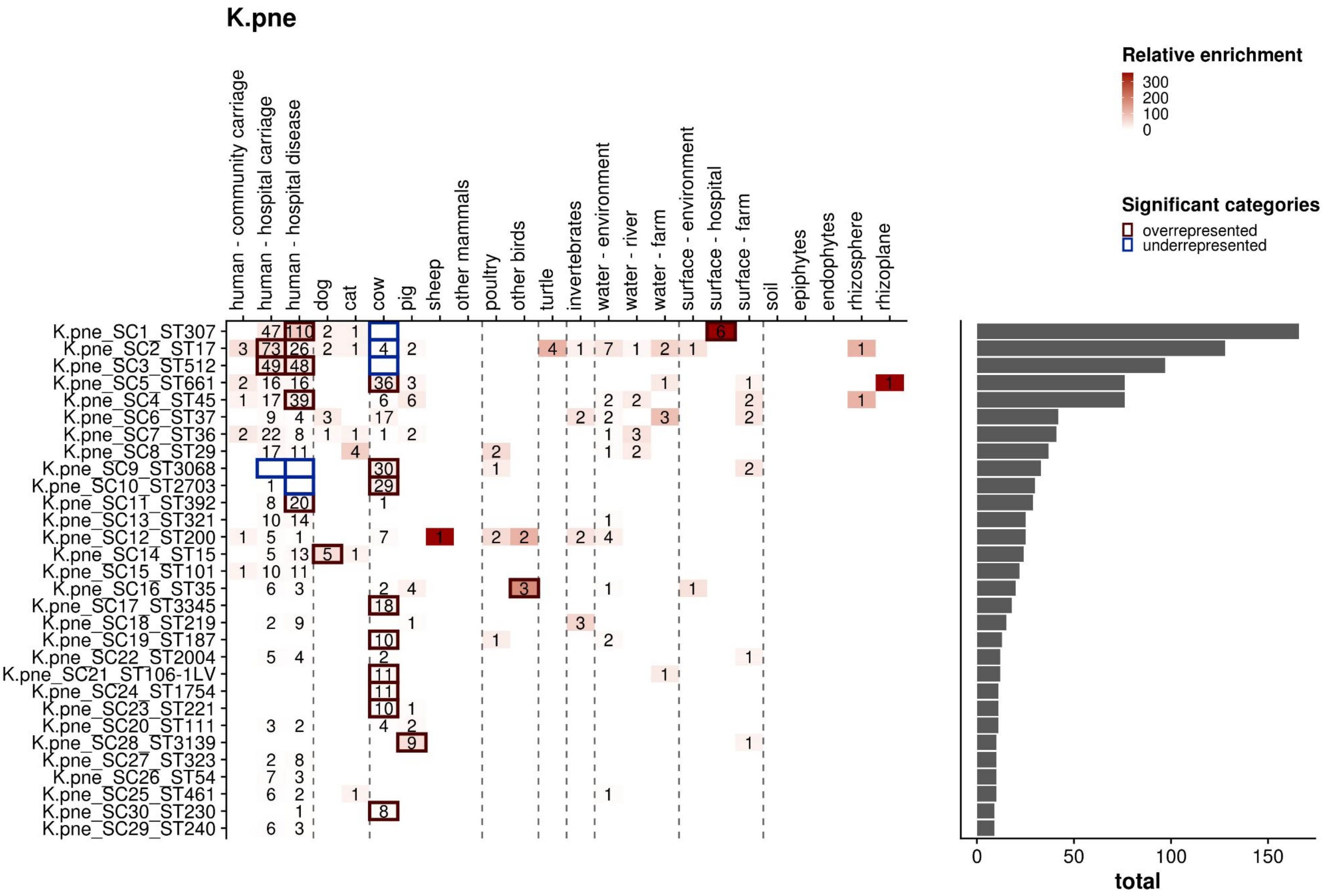


**Figure S2. The distribution of *K.pne* sequence clusters according to source.** The rows represent PopPunk SCs, and the columns represent sources delimited according to source categories. The number of positive samples are shown for each SC from each source and a blank cell indicates zero positive samples. The red shading shows the relative enrichment of each SC from each source, given the overall prevalence from that source, and assuming a null whereby all SCs would be equally likely to be observed from any given source. The dark red and blue borders show those categories where the number of samples is significantly higher or lower than expected, respectively, as determined by a permutation test. The bar plot to the right shows the number of samples from each SC.

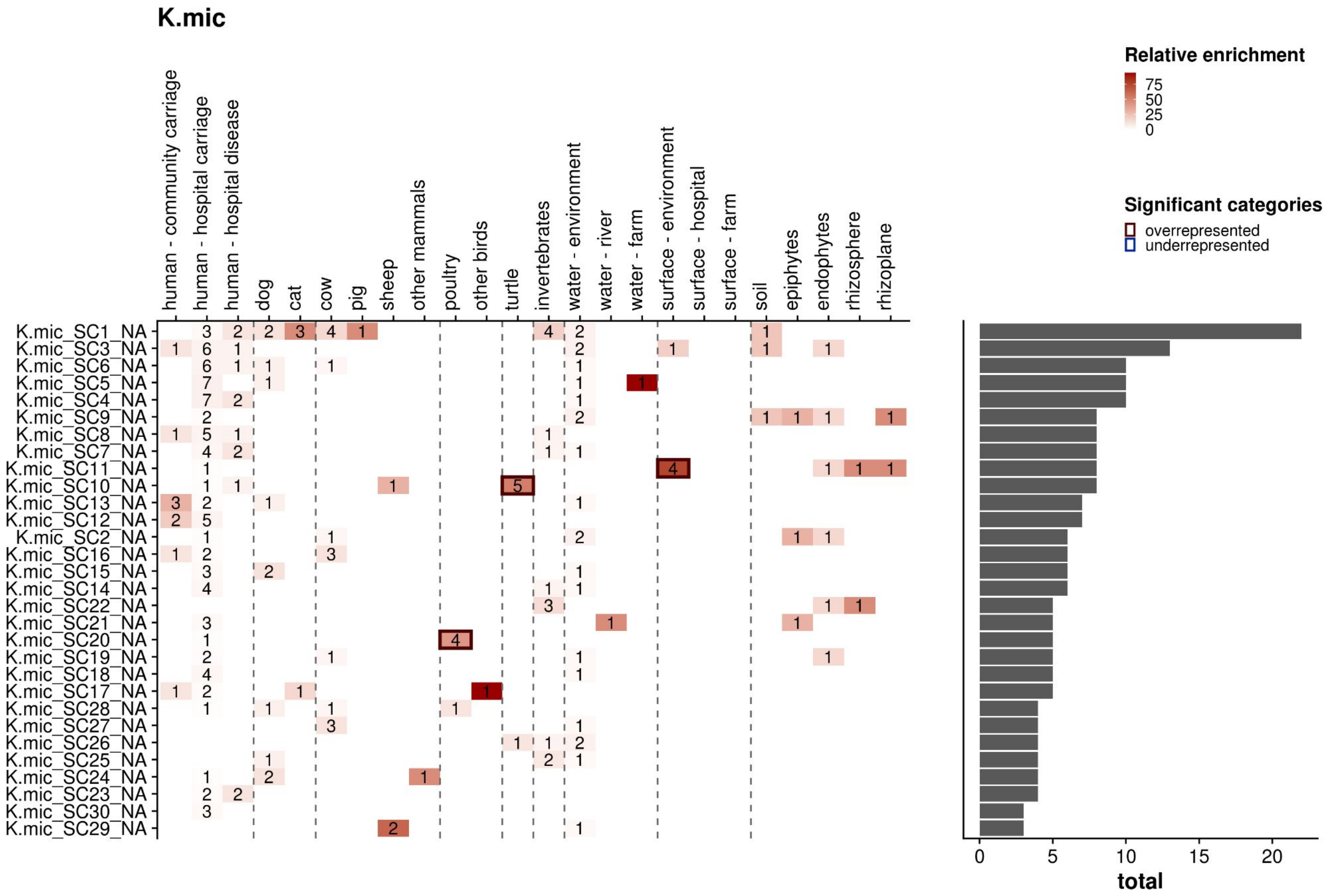


**Figure S3. The distribution of *K.mic* sequence clusters according to source.** The rows represent PopPunk SCs, and the columns represent sources delimited according to source categories. The number of positive samples are shown for each SC from each source and a blank cell indicates zero positive samples. The red shading shows the relative enrichment of each SC from each source, given the overall prevalence from that source, and assuming a null whereby all SCs would be equally likely to be observed from any given source. The dark red and blue borders show those categories where the number of samples is significantly higher or lower than expected, respectively, as determined by a permutation test. The bar plot to the right shows the number of samples from each SC.

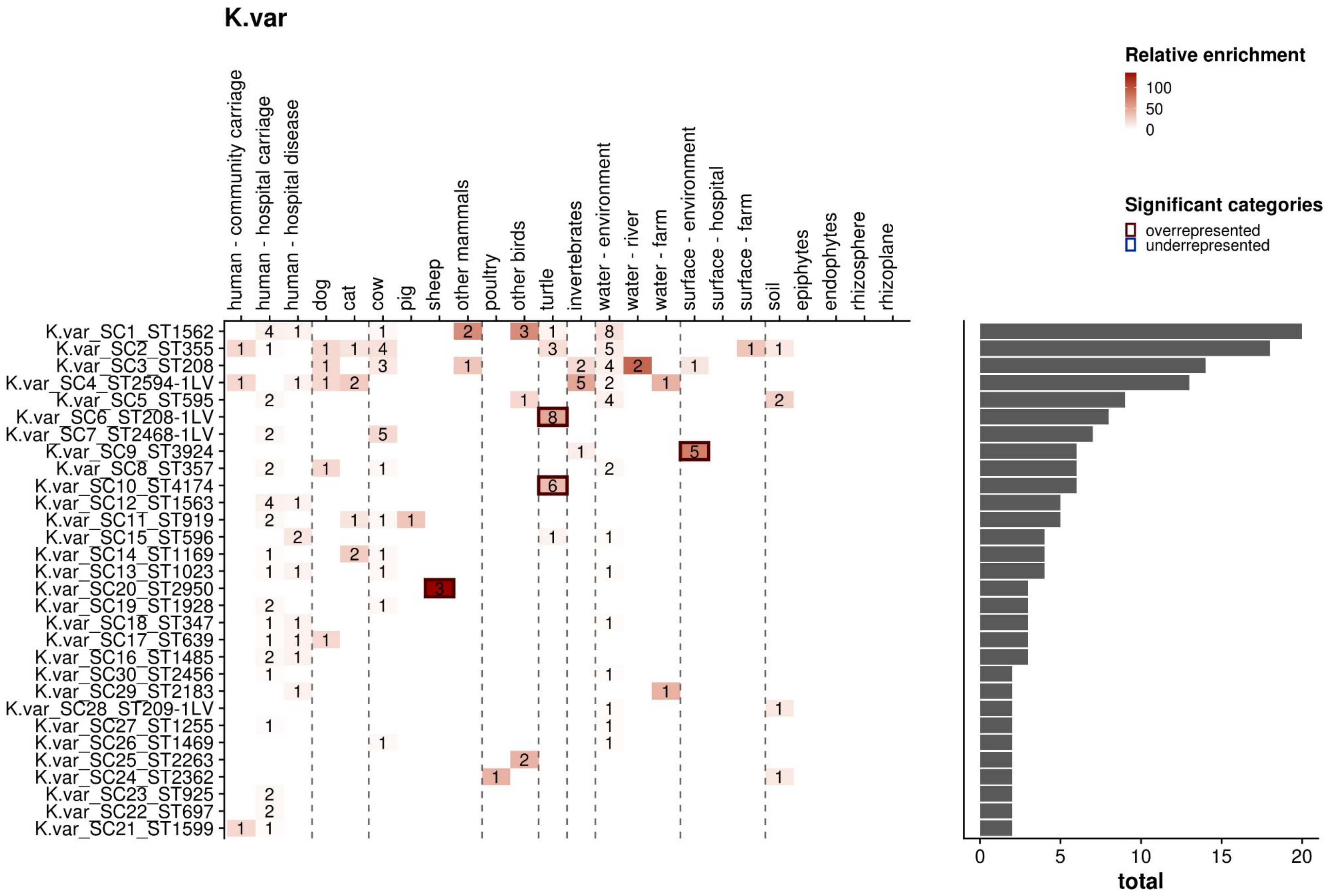


**Figure S4. The distribution of *K.var* sequence clusters according to source.** The rows represent PopPunk SCs, and the columns represent sources delimited according to source categories. The number of positive samples are shown for each SC from each source and a blank cell indicates zero positive samples. The red shading shows the relative enrichment of each SC from each source, given the overall prevalence from that source, and assuming a null whereby all SCs would be equally likely to be observed from any given source. The dark red and blue borders show those categories where the number of samples is significantly higher or lower than expected, respectively, as determined by a permutation test. The bar plot to the right shows the number of samples from each SC.

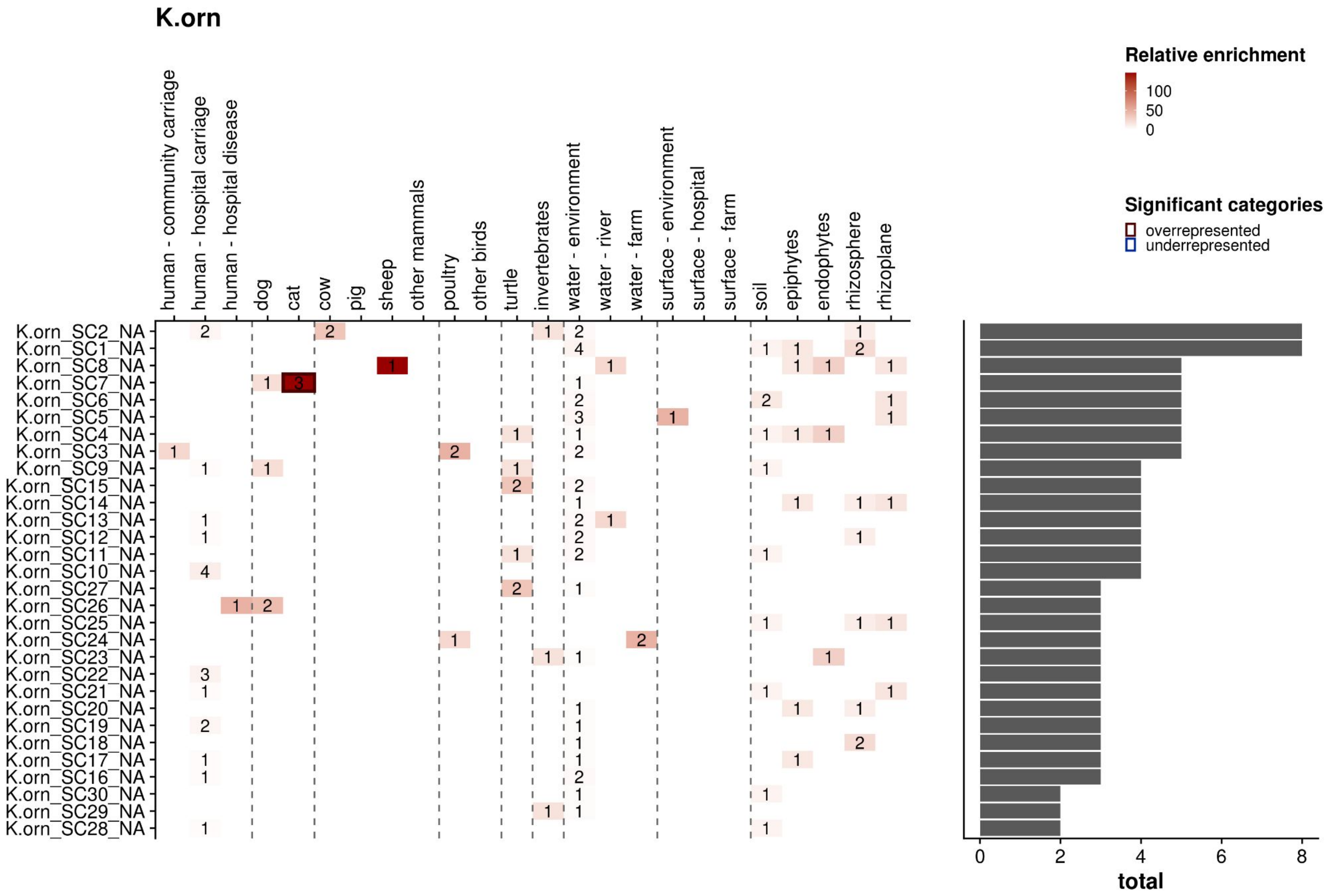


**Figure S5. The distribution of *K.orn* sequence clusters according to source.** The rows represent PopPunk SCs, and the columns represent sources delimited according to source categories. The number of positive samples are shown for each SC from each source and a blank cell indicates zero positive samples. The red shading shows the relative enrichment of each SC from each source, given the overall prevalence from that source, and assuming a null whereby all SCs would be equally likely to be observed from any given source. The dark red and blue borders show those categories where the number of samples is significantly higher or lower than expected, respectively, as determined by a permutation test. The bar plot to the right shows the number of samples from each SC.

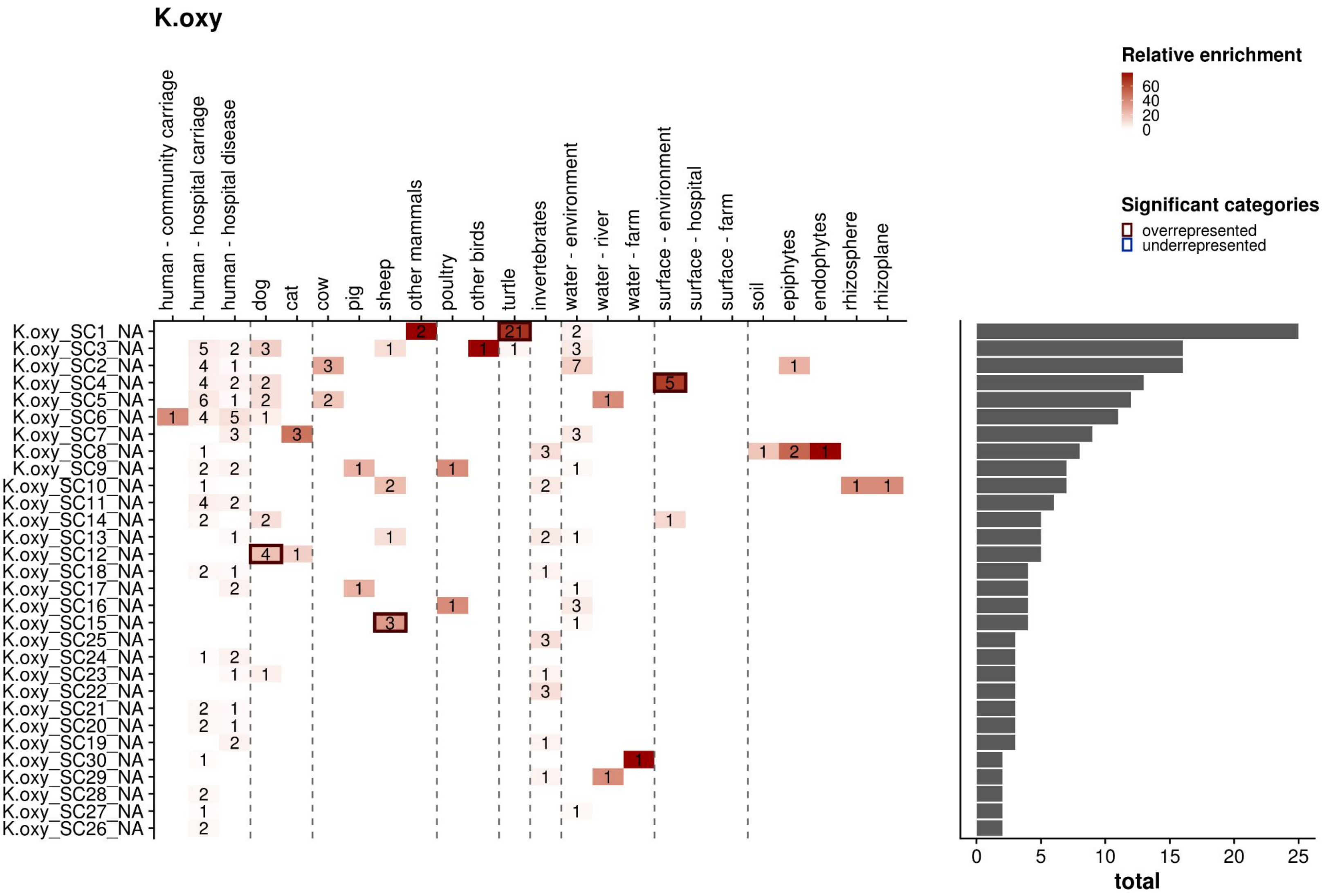


**Figure S6. The distribution of *K.oxy* sequence clusters according to source.** The rows represent PopPunk SCs, and the columns represent sources delimited according to source categories. The number of positive samples are shown for each SC from each source and a blank cell indicates zero positive samples. The red shading shows the relative enrichment of each SC from each source, given the overall prevalence from that source, and assuming a null whereby all SCs would be equally likely to be observed from any given source. The dark red and blue borders show those categories where the number of samples is significantly higher or lower than expected, respectively, as determined by a permutation test. The bar plot to the right shows the number of samples from each SC.

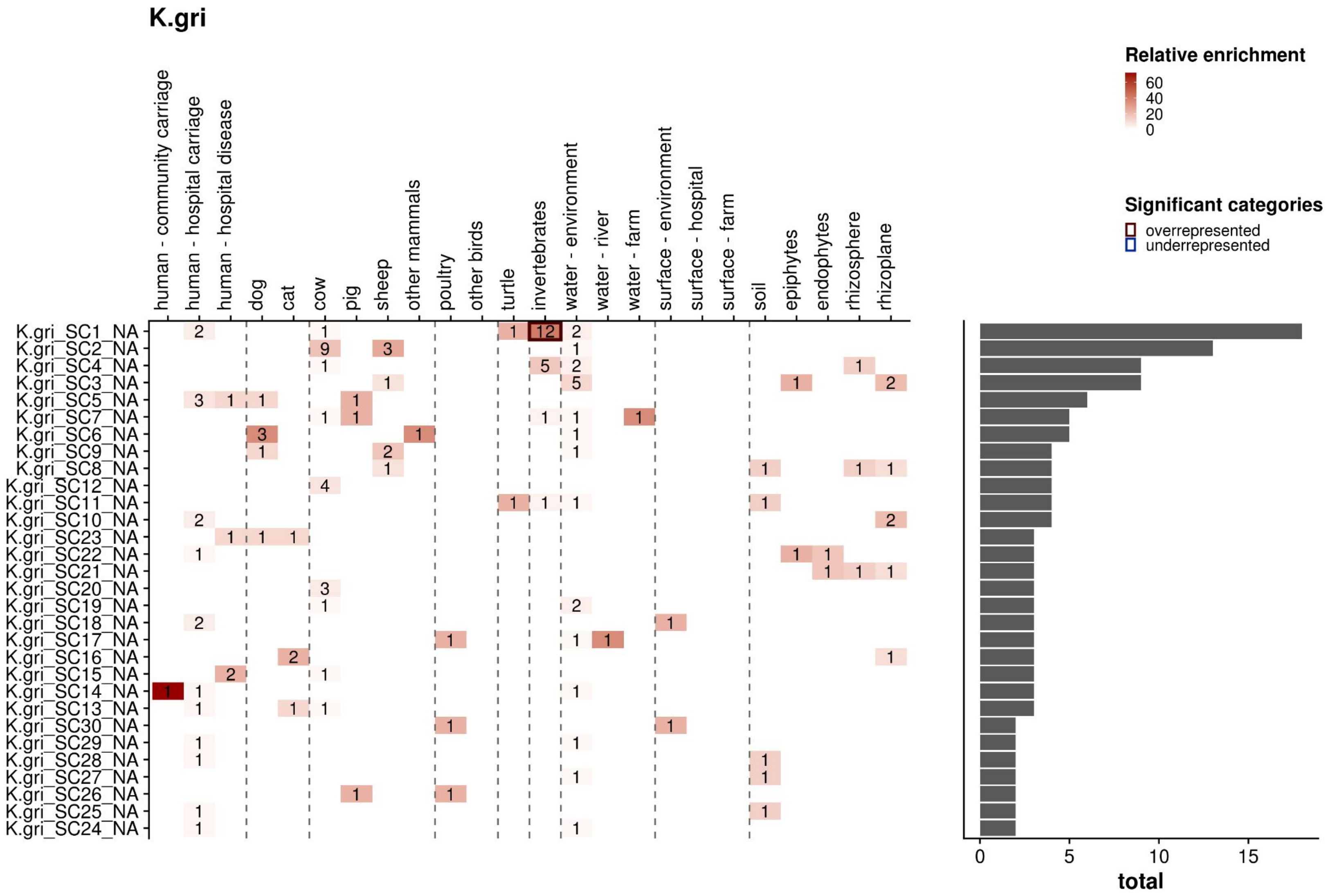


**Figure S7. The distribution of *K.gri* sequence clusters according to source.** The rows represent PopPunk SCs, and the columns represent sources delimited according to source categories. The number of positive samples are shown for each SC from each source and a blank cell indicates zero positive samples. The red shading shows the relative enrichment of each SC from each source, given the overall prevalence from that source, and assuming a null whereby all SCs would be equally likely to be observed from any given source. The dark red and blue borders show those categories where the number of samples is significantly higher or lower than expected, respectively, as determined by a permutation test. The bar plot to the right shows the number of samples from each SC.

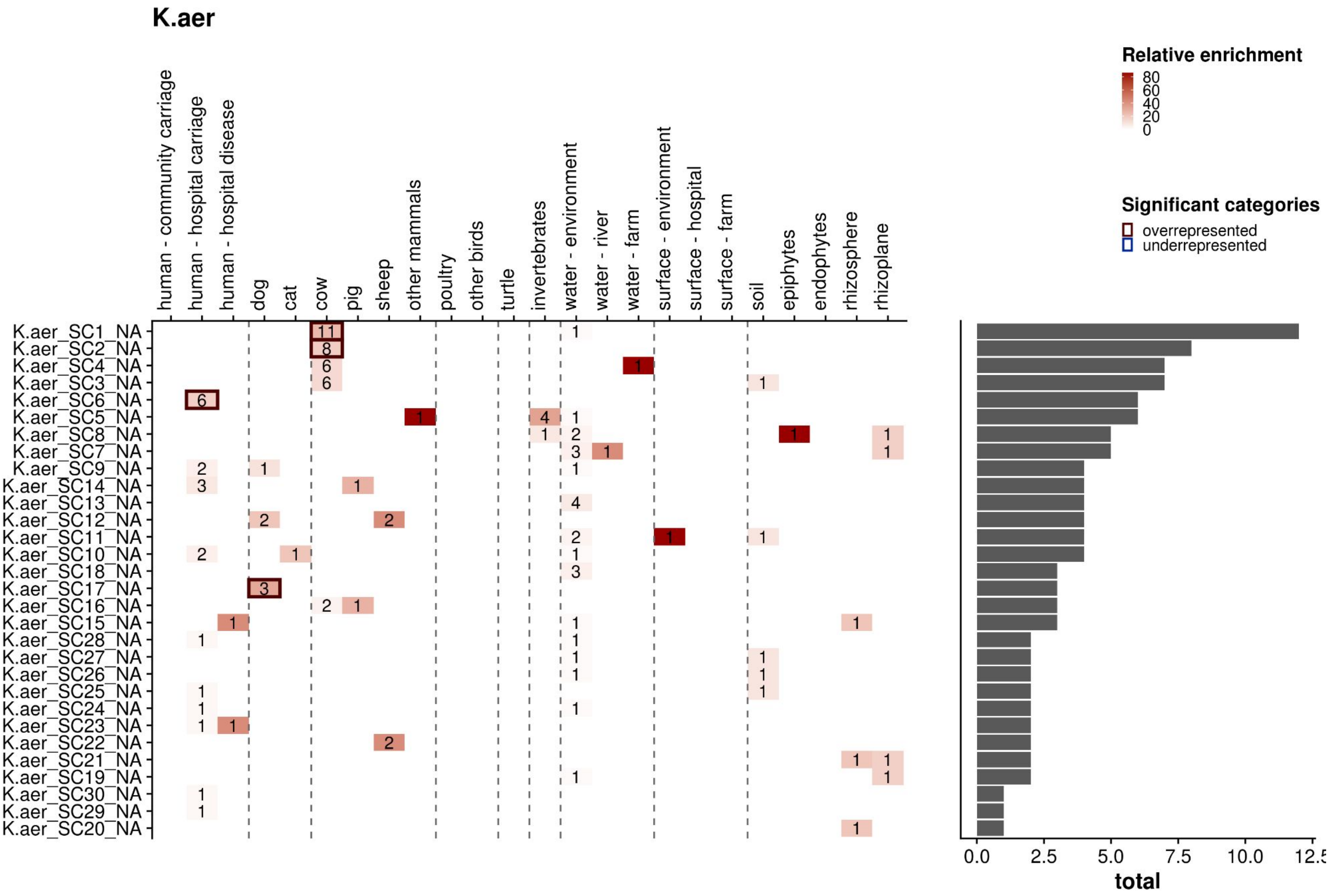


**Figure S8. The distribution of *K.aer* sequence clusters according to source.** The rows represent PopPunk SCs, and the columns represent sources delimited according to source categories. The number of positive samples are shown for each SC from each source and a blank cell indicates zero positive samples. The red shading shows the relative enrichment of each SC from each source, given the overall prevalence from that source, and assuming a null whereby all SCs would be equally likely to be observed from any given source. The dark red and blue borders show those categories where the number of samples is significantly higher or lower than expected, respectively, as determined by a permutation test. The bar plot to the right shows the number of samples from each SC.

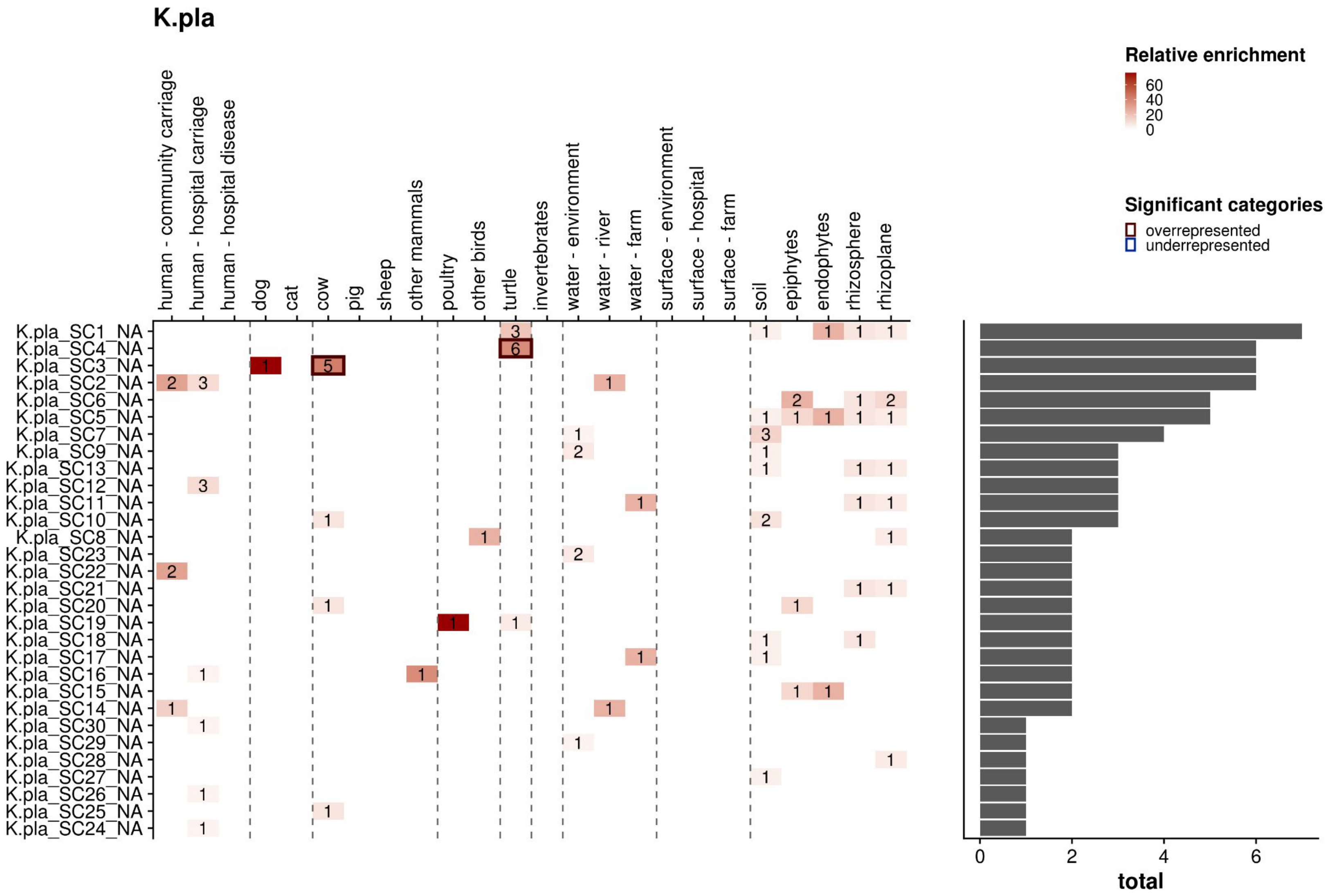


**Figure S9. The distribution of *K.pla* sequence clusters according to source.** The rows represent PopPunk SCs, and the columns represent sources delimited according to source categories. The number of positive samples are shown for each SC from each source and a blank cell indicates zero positive samples. The red shading shows the relative enrichment of each SC from each source, given the overall prevalence from that source, and assuming a null whereby all SCs would be equally likely to be observed from any given source. The dark red and blue borders show those categories where the number of samples is significantly higher or lower than expected, respectively, as determined by a permutation test. The bar plot to the right shows the number of samples from each SC.

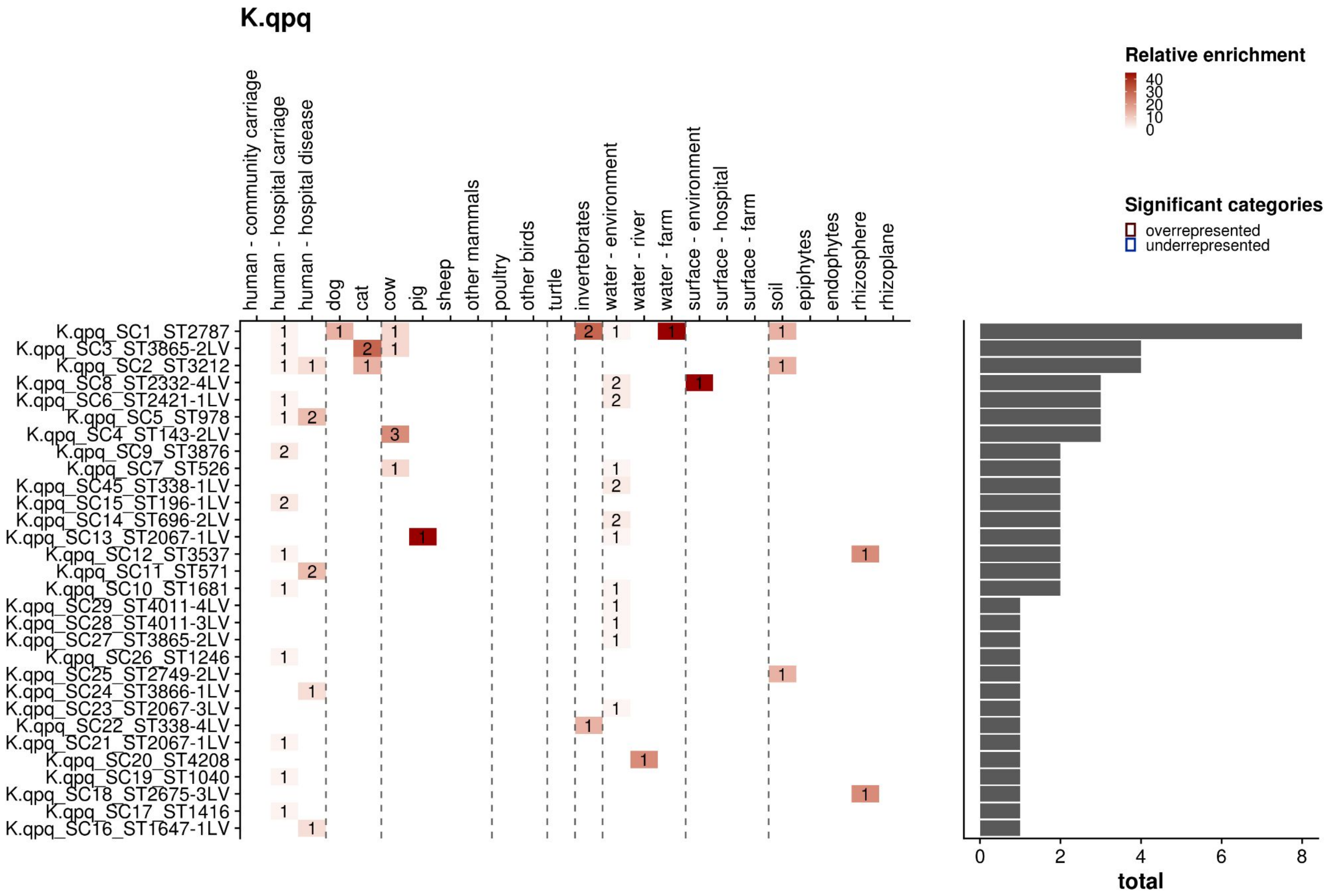


**Figure S10. The distribution of *K.qpq* sequence clusters according to source.** The rows represent PopPunk SCs, and the columns represent sources delimited according to source categories. The number of positive samples are shown for each SC from each source and a blank cell indicates zero positive samples. The red shading shows the relative enrichment of each SC from each source, given the overall prevalence from that source, and assuming a null whereby all SCs would be equally likely to be observed from any given source. The dark red and blue borders show those categories where the number of samples is significantly higher or lower than expected, respectively, as determined by a permutation test. The bar plot to the right shows the number of samples from each SC.

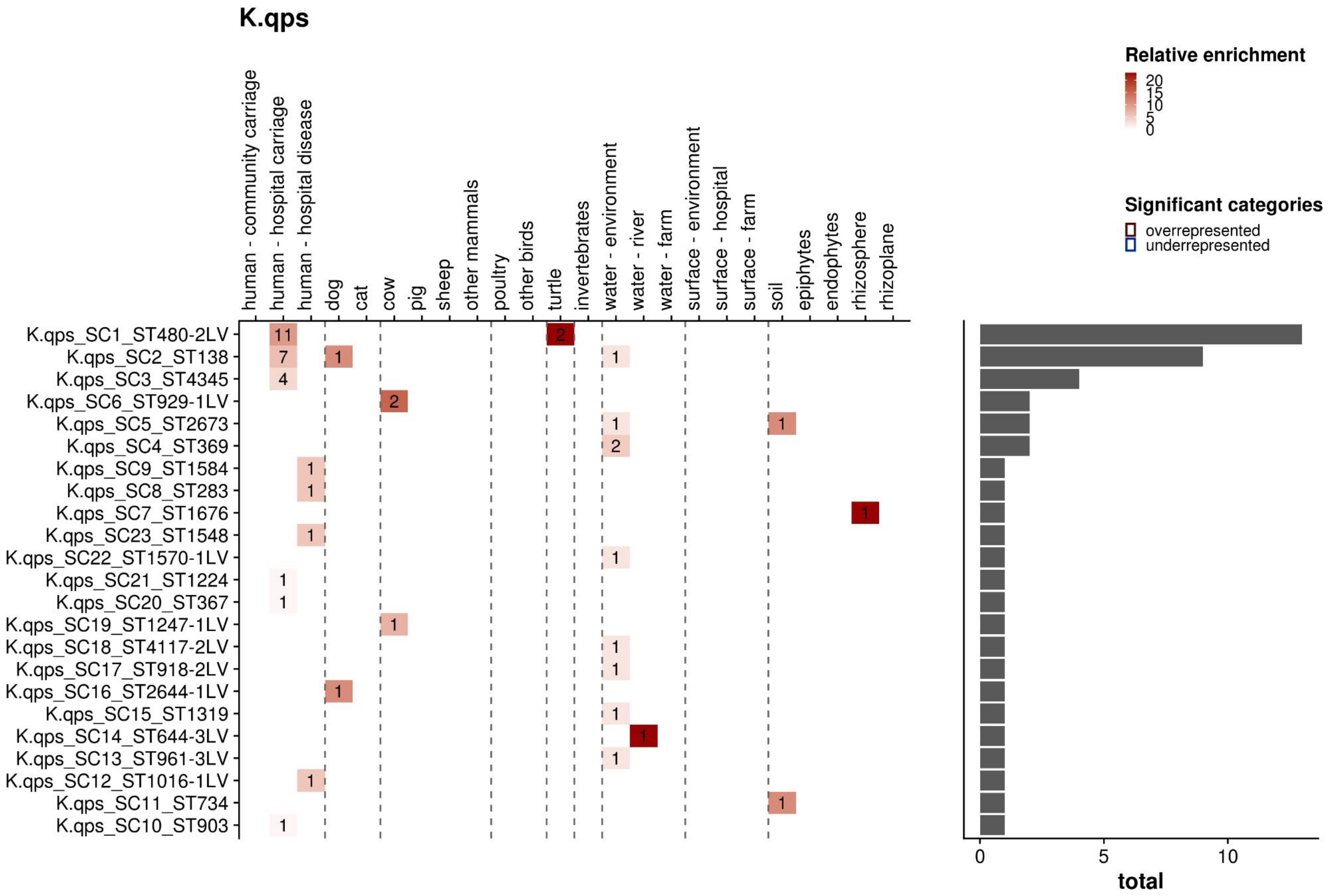


**Figure S11. The distribution of *K.qps* sequence clusters according to source.** The rows represent PopPunk SCs, and the columns represent sources delimited according to source categories. The number of positive samples are shown for each SC from each source and a blank cell indicates zero positive samples. The red shading shows the relative enrichment of each SC from each source, given the overall prevalence from that source, and assuming a null whereby all SCs would be equally likely to be observed from any given source. The dark red and blue borders show those categories where the number of samples is significantly higher or lower than expected, respectively, as determined by a permutation test. The bar plot to the right shows the number of samples from each SC.

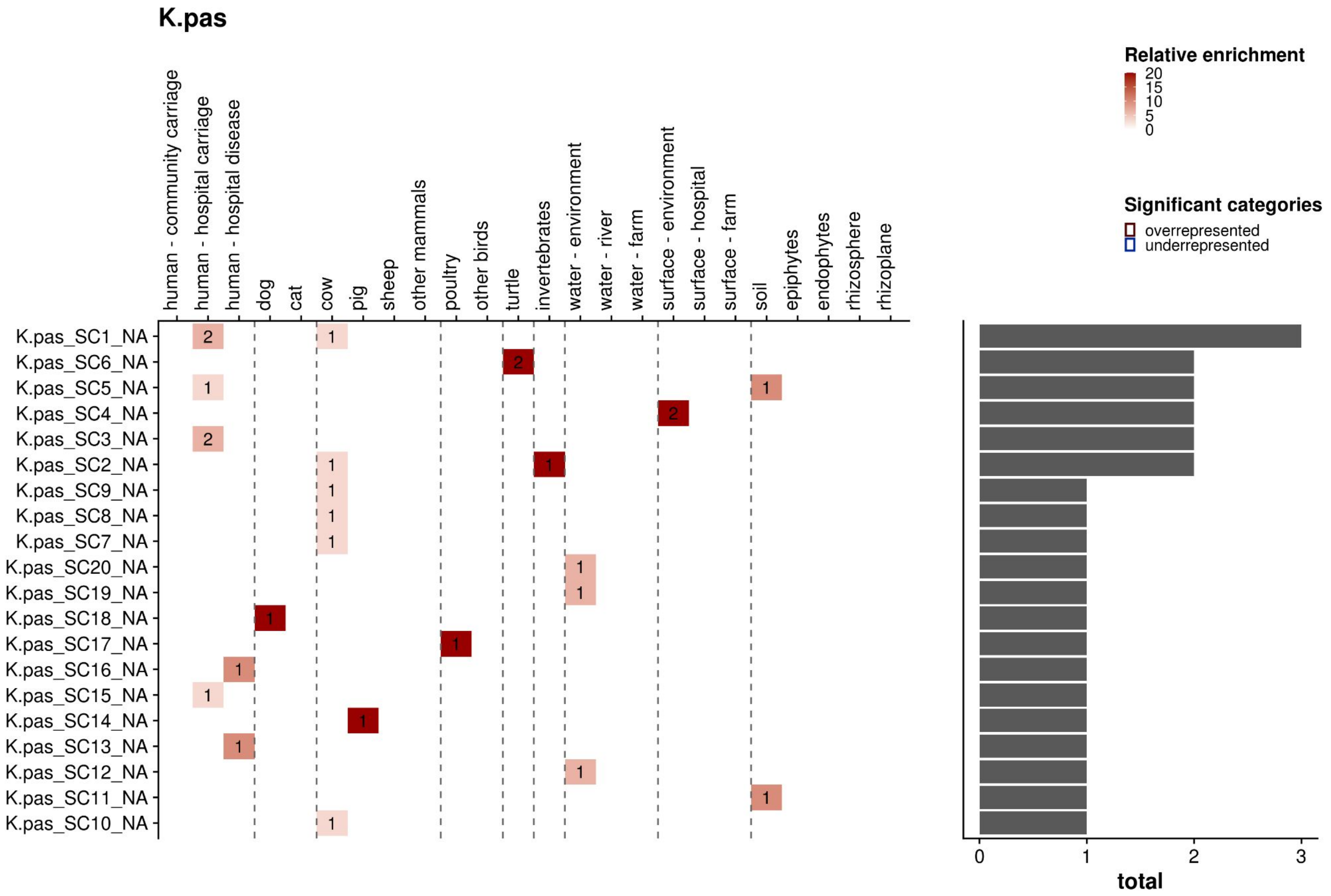


**Figure S12. The distribution of *K.pas* sequence clusters according to source.** The rows represent PopPunk SCs, and the columns represent sources delimited according to source categories. The number of positive samples are shown for each SC from each source and a blank cell indicates zero positive samples. The red shading shows the relative enrichment of each SC from each source, given the overall prevalence from that source, and assuming a null whereby all SCs would be equally likely to be observed from any given source. The dark red and blue borders show those categories where the number of samples is significantly higher or lower than expected, respectively, as determined by a permutation test. The bar plot to the right shows the number of samples from each SC.

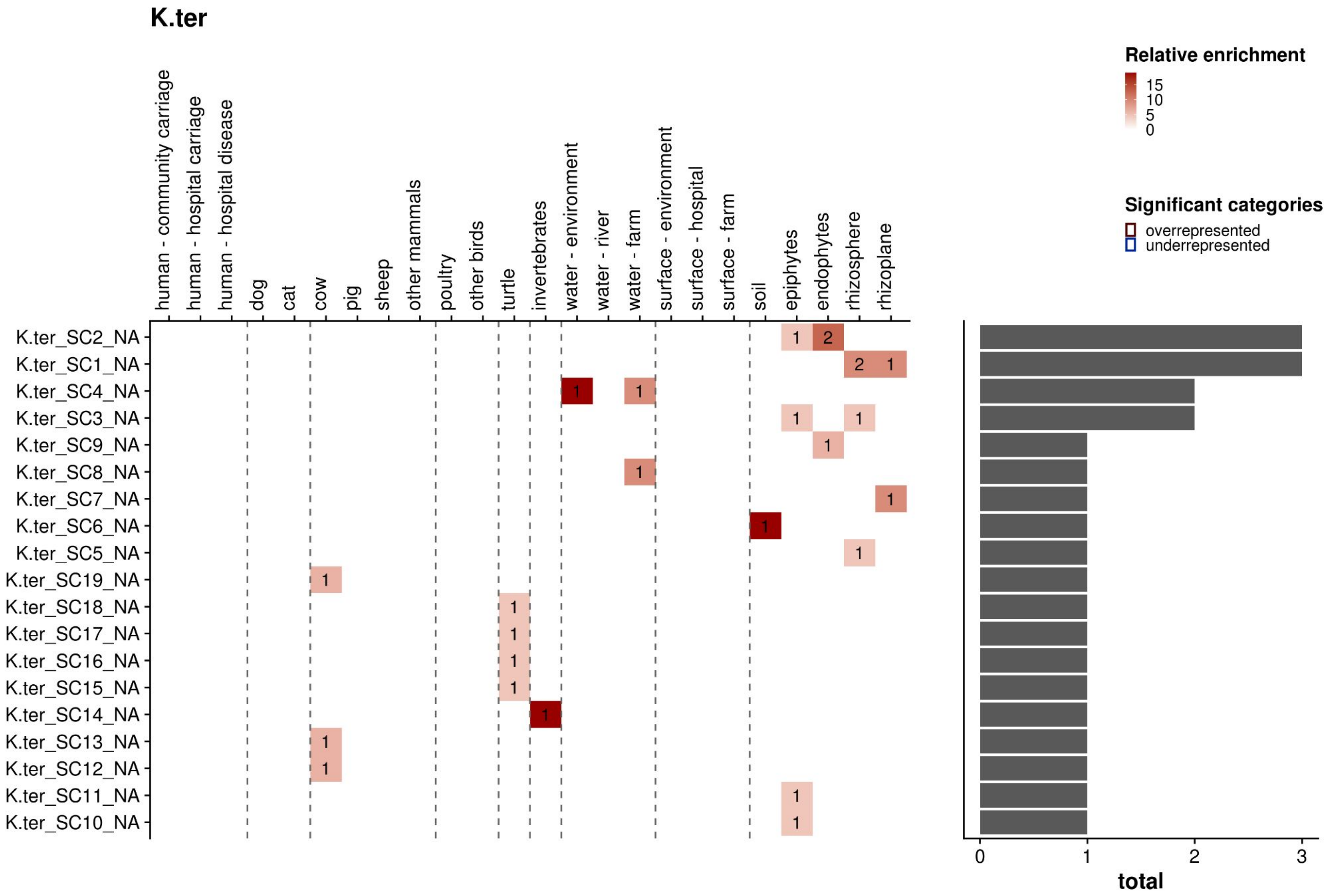


**Figure S13. The distribution of *K.ter* sequence clusters according to source.** The rows represent PopPunk SCs, and the columns represent sources delimited according to source categories. The number of positive samples are shown for each SC from each source and a blank cell indicates zero positive samples. The red shading shows the relative enrichment of each SC from each source, given the overall prevalence from that source, and assuming a null whereby all SCs would be equally likely to be observed from any given source. The dark red and blue borders show those categories where the number of samples is significantly higher or lower than expected, respectively, as determined by a permutation test. The bar plot to the right shows the number of samples from each SC.

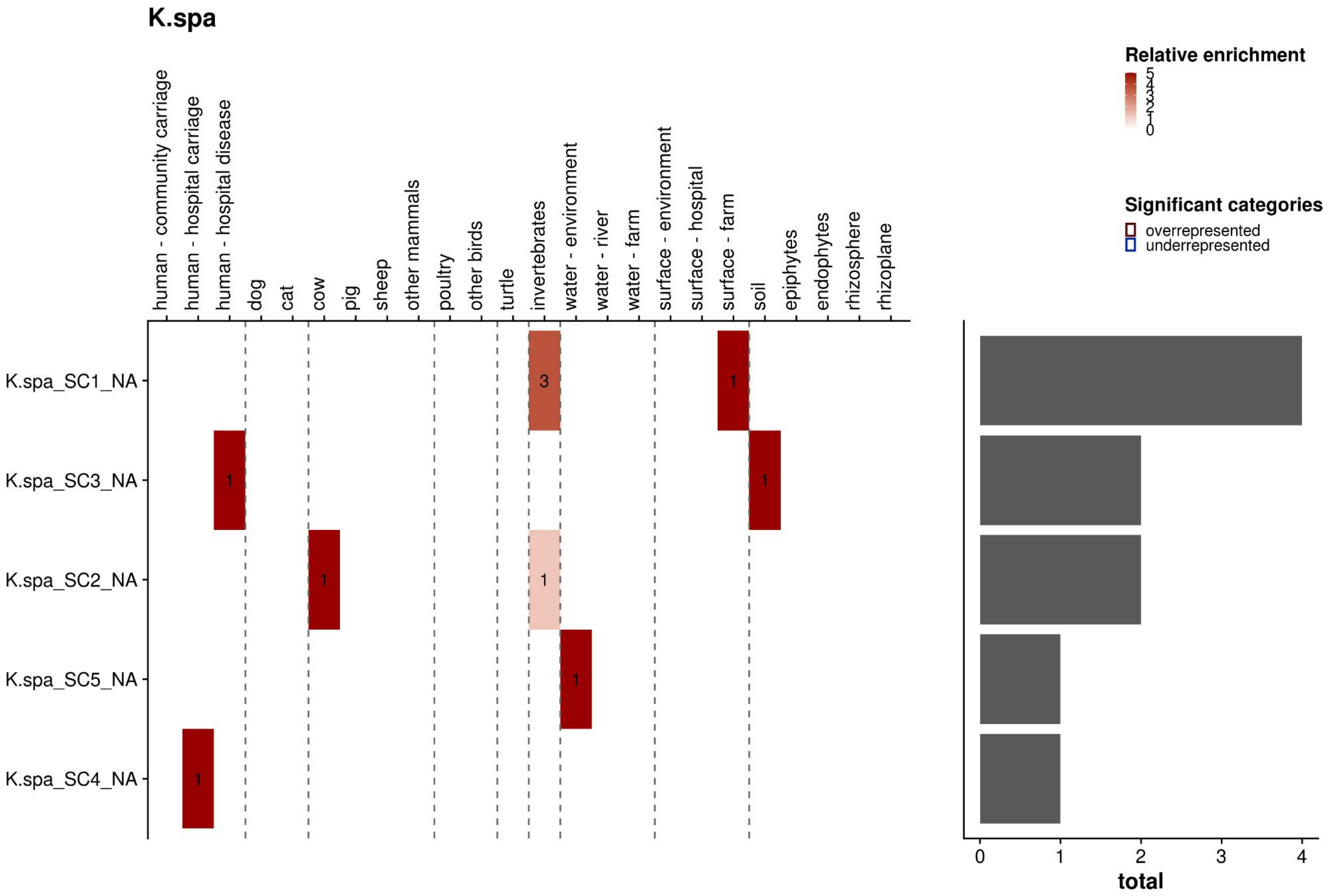


**Figure S14. The distribution of *K.spa* sequence clusters according to source.** The rows represent PopPunk SCs, and the columns represent sources delimited according to source categories. The number of positive samples are shown for each SC from each source and a blank cell indicates zero positive samples. The red shading shows the relative enrichment of each SC from each source, given the overall prevalence from that source, and assuming a null whereby all SCs would be equally likely to be observed from any given source. The dark red and blue borders show those categories where the number of samples is significantly higher or lower than expected, respectively, as determined by a permutation test. The bar plot to the right shows the number of samples from each SC.

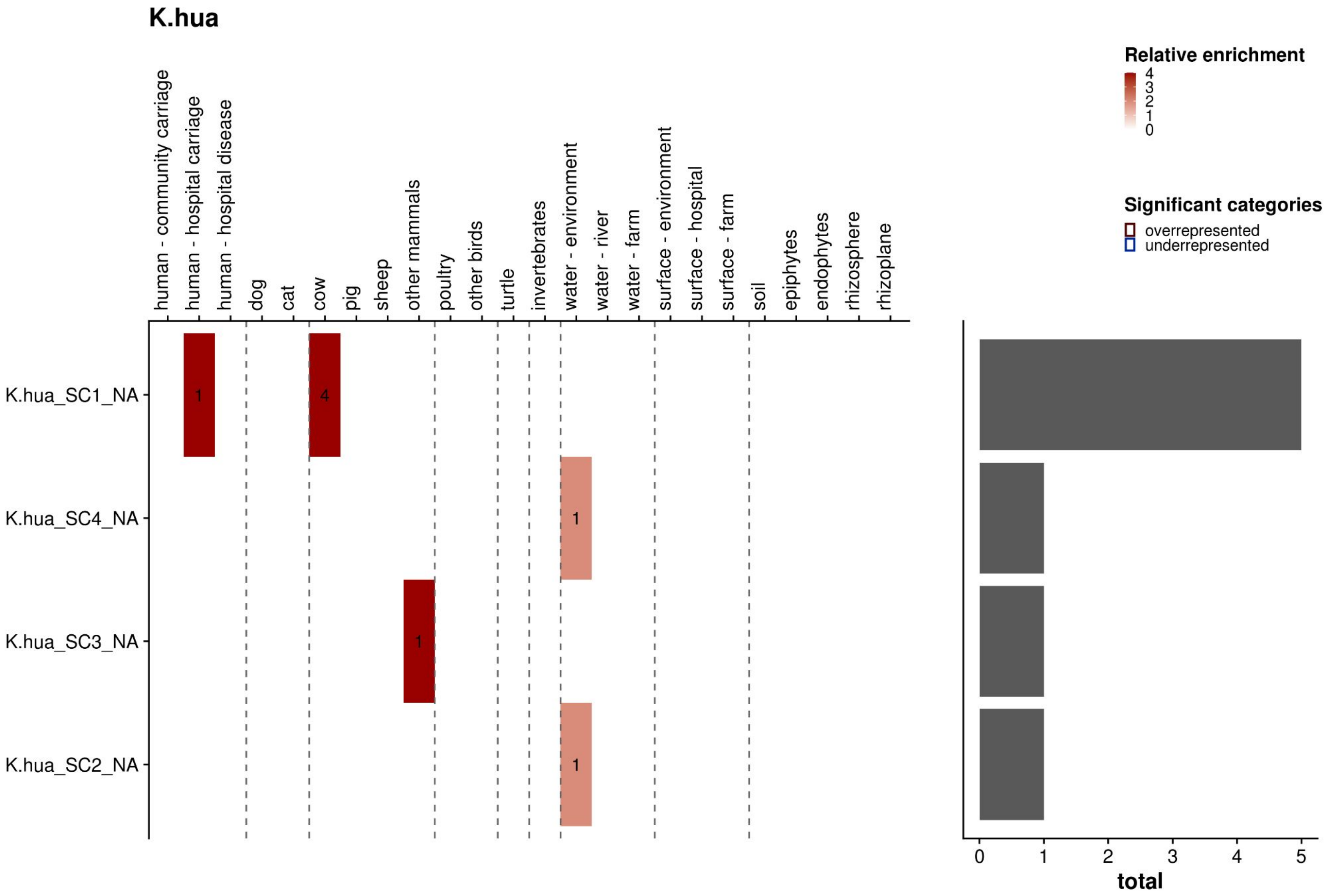


**Figure S15. The distribution of *K.hua* sequence clusters according to source.** The rows represent PopPunk SCs, and the columns represent sources delimited according to source categories. The number of positive samples are shown for each SC from each source and a blank cell indicates zero positive samples. The red shading shows the relative enrichment of each SC from each source, given the overall prevalence from that source, and assuming a null whereby all SCs would be equally likely to be observed from any given source. The dark red and blue borders show those categories where the number of samples is significantly higher or lower than expected, respectively, as determined by a permutation test. The bar plot to the right shows the number of samples from each SC.

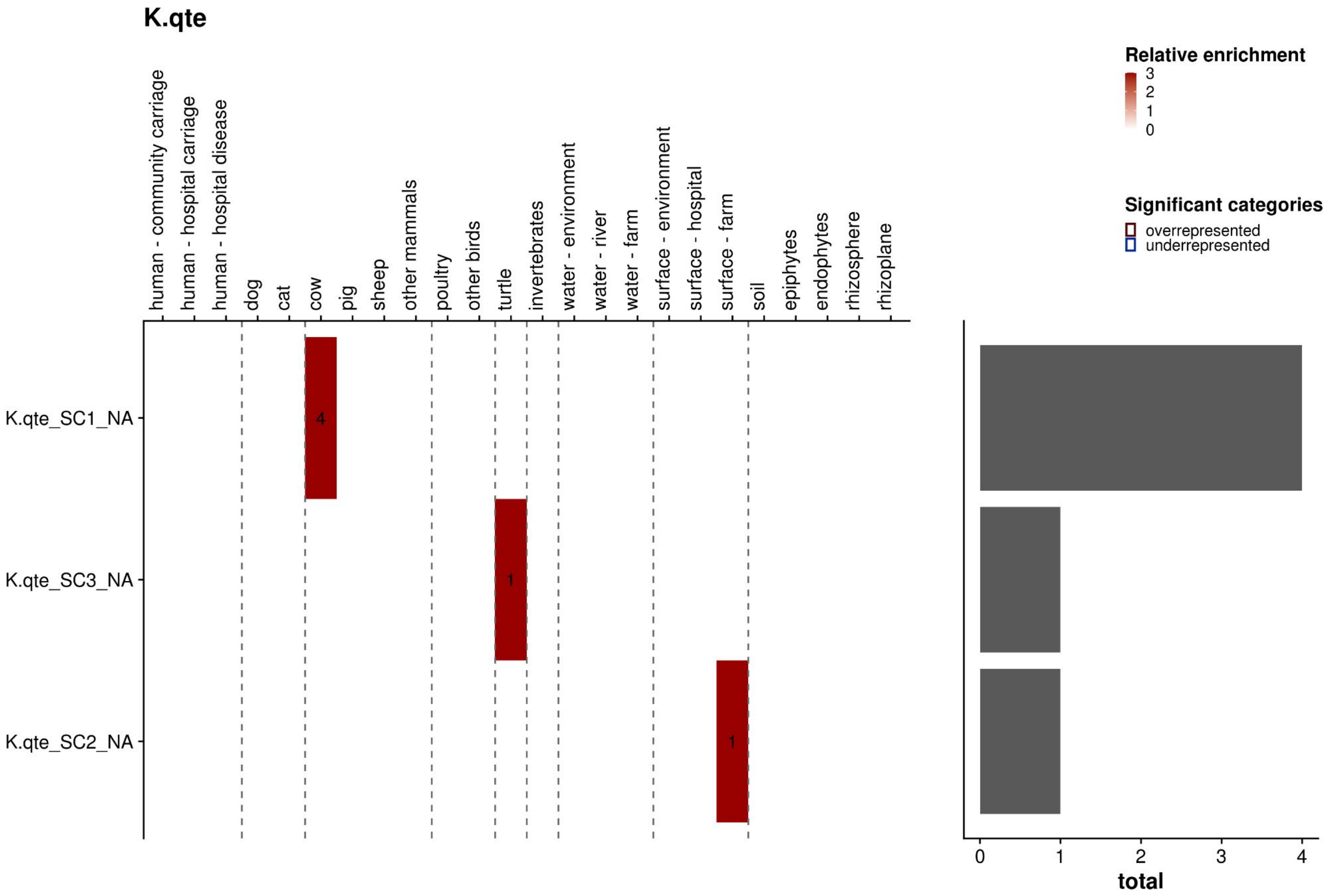


**Figure S16. The distribution of *K.qte* sequence clusters according to source.** The rows represent PopPunk SCs, and the columns represent sources delimited according to source categories. The number of positive samples are shown for each SC from each source and a blank cell indicates zero positive samples. The red shading shows the relative enrichment of each SC from each source, given the overall prevalence from that source, and assuming a null whereby all SCs would be equally likely to be observed from any given source. The dark red and blue borders show those categories where the number of samples is significantly higher or lower than expected, respectively, as determined by a permutation test. The bar plot to the right shows the number of samples from each SC.

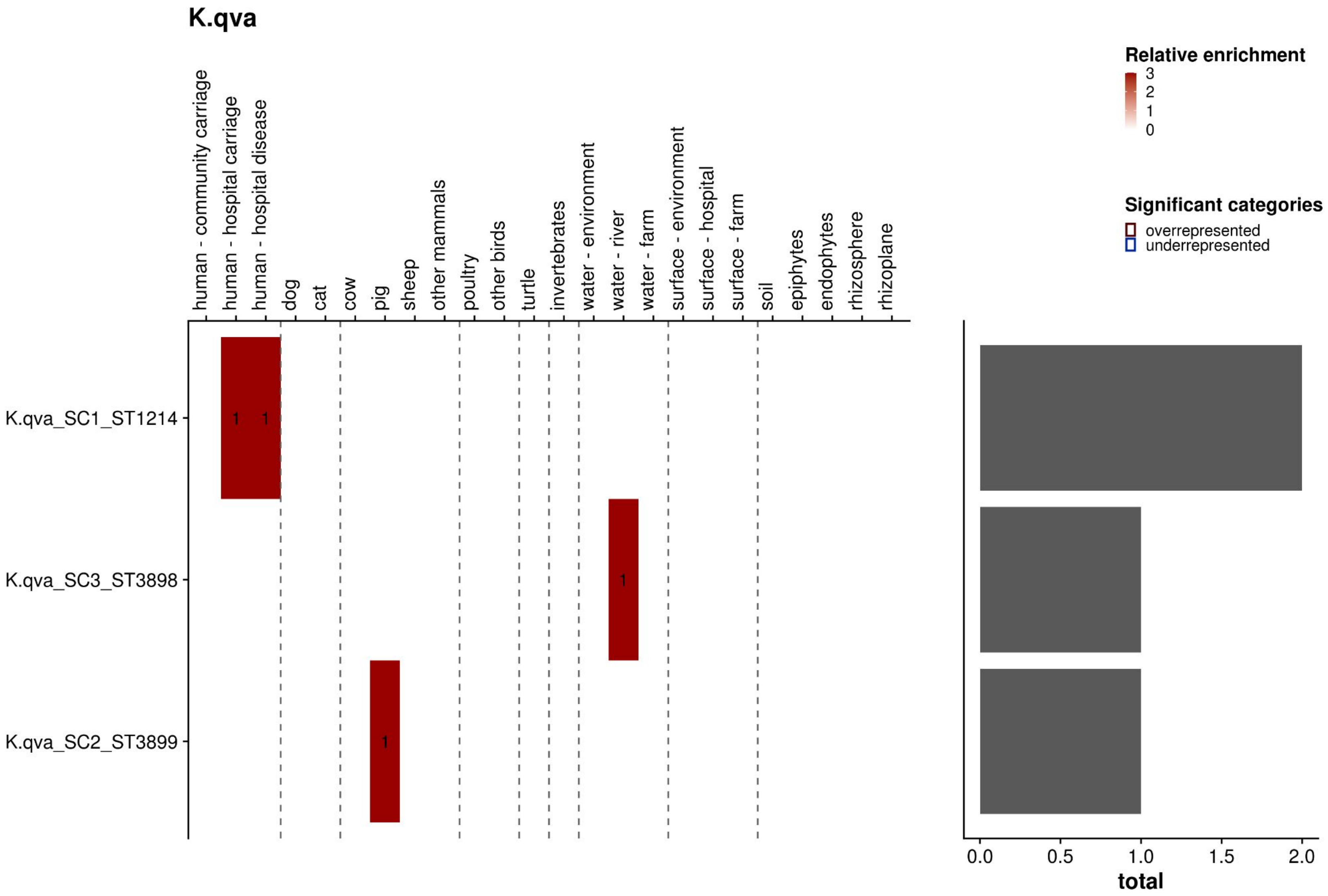


**Figure S17. The distribution of *K.qva* sequence clusters according to source.** The rows represent PopPunk SCs, and the columns represent sources delimited according to source categories. The number of positive samples are shown for each SC from each source and a blank cell indicates zero positive samples. The red shading shows the relative enrichment of each SC from each source, given the overall prevalence from that source, and assuming a null whereby all SCs would be equally likely to be observed from any given source. The dark red and blue borders show those categories where the number of samples is significantly higher or lower than expected, respectively, as determined by a permutation test. The bar plot to the right shows the number of samples from each SC.

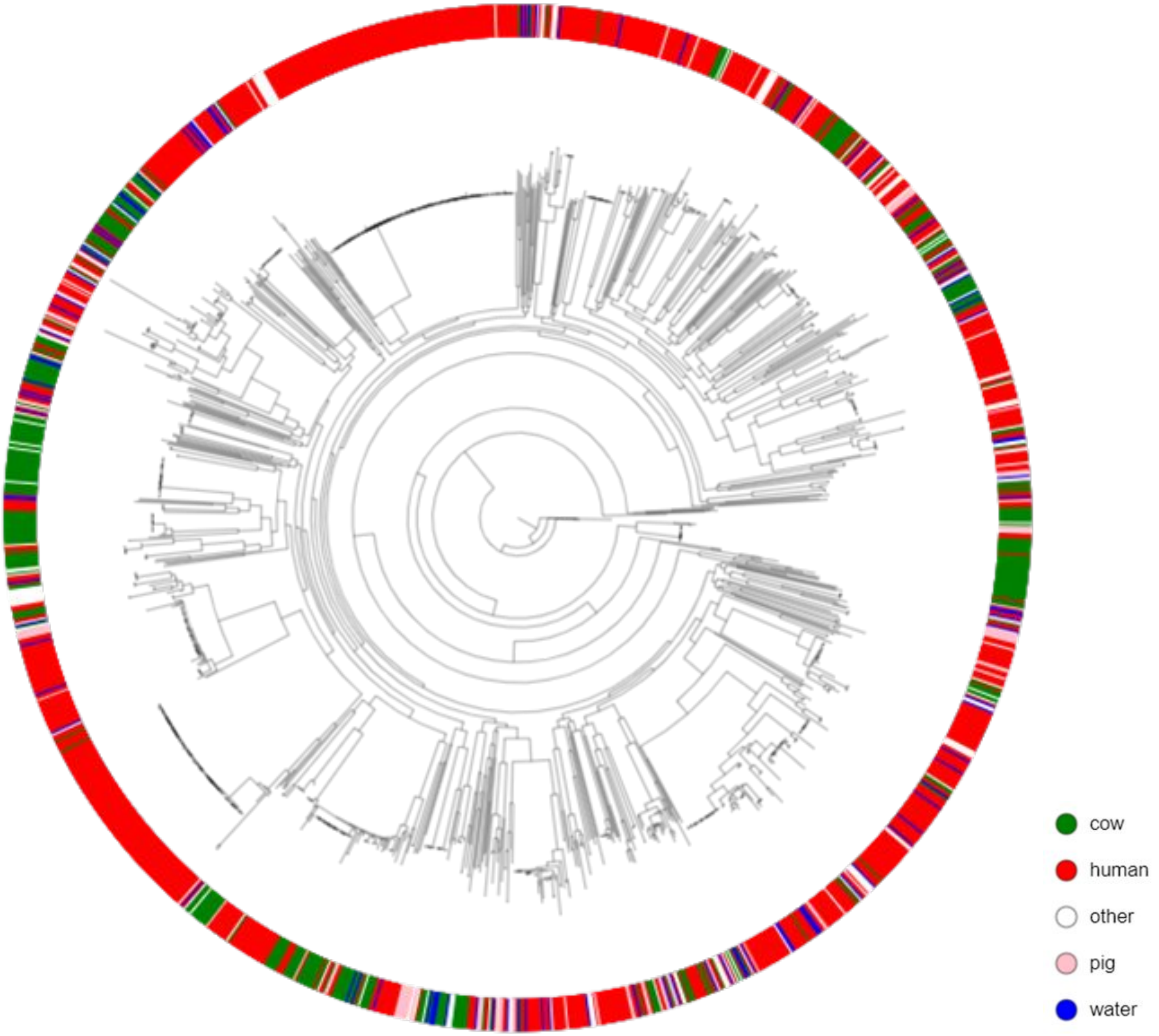


**Figure S18. Phylogenetic analysis of *K.pne* isolates.** Human (red) and cow (green) isolates of *K.pne* are non-randomly distributed across the phylogenetic tree.

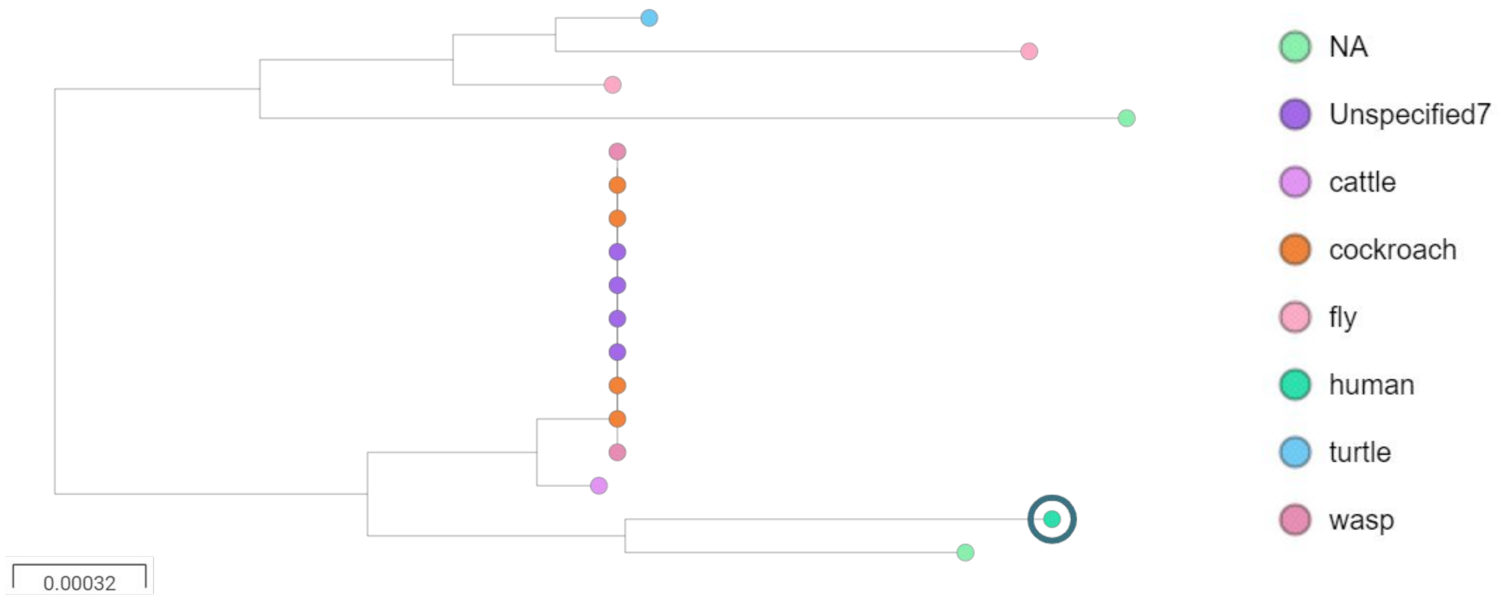


**Figure S19. *K.gri* SC1 isolates.** Most isolates are associated with invertebrates, and the only isolate from a hospital outpatient is ringed in the tree.
