# Supplementary material for: A large-scale genomic snapshot of Klebsiella spp. isolates in Northern Italy reveals limited transmission between clinical and non-clinical settings
Source: Nat Microbiol. 2022 Nov 21;7(12):2054–67. doi: 10.1038/s41564-022-01263-0 (PMC9712112; doi:10.1038/s41564-022-01263-0)
Supplement: Supplementary file 2 — Reporting Summary [file 41564_2022_1263_MOESM2_ESM.pdf]

nature portfolio

Corresponding author(s): Edward Feil

Last updated by author(s): Sep 22, 2022

Reporting Summary

Nature Portfolio wishes to improve the reproducibility of the work that we publish. This form provides structure for consistency and transparency in reporting. For further information on Nature Portfolio policies, see our Editorial Policies and the Editorial Policy Checklist.

Statistics

For all statistical analyses, confirm that the following items are present in the figure legend, table legend, main text, or Methods section.

n/a	Confirmed	
☐	☒	The exact sample size (*n*) for each experimental group/condition, given as a discrete number and unit of measurement
☒	☐	A statement on whether measurements were taken from distinct samples or whether the same sample was measured repeatedly
☐	☒	The statistical test(s) used AND whether they are one- or two-sided<br>*Only common tests should be described solely by name; describe more complex techniques in the Methods section.*
☒	☐	A description of all covariates tested
☒	☐	A description of any assumptions or corrections, such as tests of normality and adjustment for multiple comparisons
☒	☐	A full description of the statistical parameters including central tendency (e.g. means) or other basic estimates (e.g. regression coefficient) AND variation (e.g. standard deviation) or associated estimates of uncertainty (e.g. confidence intervals)
☐	☒	For null hypothesis testing, the test statistic (e.g. *F*, *t*, *r*) with confidence intervals, effect sizes, degrees of freedom and *P* value Noted. *Give P values as exact values whenever suitable.*
☒	☐	For Bayesian analysis, information on the choice of priors and Markov chain Monte Carlo settings
☒	☐	For hierarchical and complex designs, identification of the appropriate level for tests and full reporting of outcomes
☒	☐	Estimates of effect sizes (e.g. Cohen's *d*, Pearson's *r*), indicating how they were calculated

Our web collection on statistics for biologists contains articles on many of the points above.

Software and code

Policy information about availability of computer code

Data collection	Excel 365 was used to compile and record the list of samples as they were being generated
Data analysis	Bruker Biotyper 3.1 was used to interpret the data from Maldi-ToF for species ID<br>Trimmomatic v. 0.33 was used to trim the Illumina reads<br>SPAdes v3.9.0 was used to generated de novo assemblies<br>Prokka v1.12 was used to annotate the genome assemblies, and Prokka v1.4.5 was used to annotate the plamids<br>Kleborate v2.0.0 was used to assign species and ST (and call resistance and virulence genes) from the assemblies<br>PopPunk v2.0.2 was used to delimit sequence clusters (SCs)<br>Snippy v4.6.0 was used for mapping<br>Abricate v1.0.0 was used to assign AMR genes to contigs<br>RapidNJ, (no version informaion available), and RaXML v8.2.8 were used for phylogenetic analysis<br>R code for the transmission analysis and permutation tests has been deposited in github:<br>https://github.com/harry-thorpe/SPARK_code

For manuscripts utilizing custom algorithms or software that are central to the research but not yet described in published literature, software must be made available to editors and reviewers. We strongly encourage code deposition in a community repository (e.g. GitHub). See the Nature Portfolio guidelines for submitting code & software for further information.

Data

Policy information about availability of data

All manuscripts must include a data availability statement. This statement should provide the following information, where applicable:

- Accession codes, unique identifiers, or web links for publicly available datasets
- A description of any restrictions on data availability
- For clinical datasets or third party data, please ensure that the statement adheres to our policy

Data availability: Short-read data available under accession numbers ERR3412430 to ERR3412448;ERR3440341 to ERR3440427;ERR3448863 to ERR3449598;ERR3469775 to ERR3469909;ERR3479903 to ERR3480717;ERR3844616 to ERR3844777;ERR3904469 to ERR3904709;ERR3931787 to ERR3932313;ERR3967745 to ERR3967936;ERR4022833 to ERR4023150;ERR4139181 to ERR4139191;ERR4374646 to ERR4374837.
The metadata and tree are avilable to download form the Microreact project at https://microreact.org/project/KLEBPAVIA.
PlasmidFinder and Resfinder databases downloaded on 21/05/2021 from https://bitbucket.org/genomicepidemiology/plasmidfinder_db and https://bitbucket.org/genomicepidemiology/resfinder_db, respectively.
Map tiles by Stamen Design, under CC BY 3.0. Data by OpenStreetMap, under ODbL.

Field-specific reporting

Please select the one below that is the best fit for your research. If you are not sure, read the appropriate sections before making your selection.

☐ Life sciences ☐ Behavioural & social sciences ☒ Ecological, evolutionary & environmental sciences

For a reference copy of the document with all sections, see nature.com/documents/nr-reporting-summary-flat.pdf

Ecological, evolutionary & environmental sciences study design

All studies must disclose on these points even when the disclosure is negative.

Study description	The study focussed on the characterisation -by whole-genome sequencing- of a large collection of bacteria within to the genus Klebsiella. The aim was to examine the likely role of one-health reservoirs of Klebsiella in the emergence and spread of antimicrobial resistance.
Research sample	The Klebsiella isolates were sampled from multiple clinical, community, animal and environmental settings. Animal samples were from swabs only.
Sampling strategy	Most isolates were recovered by culturing on selective media (SCAI). Clinical isolates were acquired as part of ongoing surveillance projects in the Pavia region. The final sample size (3482 complete genomes) is one of the largest studies of its kind and is sufficient to draw inferences regarding transmission frequencies between different settings. No size calculation was carried out as we were not aiming to capture all the diversity within these populations.
Data collection	Metadata (place, time, source information) pertaining to all the samples (both positive and negative) were recorded by members of the team in Pavia. The sequence data was generated on the Illumina platform at the Wellcome Trust Sanger Institute in Cambridge, UK.
Timing and spatial scale	The data were collected in and around the Northern Italian city of Pavia between June 2017 and November 2018.
Data exclusions	A small number of genome sequences were excluded on the basis that they did not pass QC.
Reproducibility	Multiple samples were taken from each source
Randomization	The samples were grouped by source (eg human, cow, river water, rhizosphere etc). These are discrete categories.
Blinding	The isolates were cultured without selecting for distinct species or resistance profile. Species ID by WGS was derived Independently from species ID by Maldi-ToF.

Did the study involve field work? ☒ Yes ☐ No

Field work, collection and transport

Field conditions	Environmental parameters such as temperature and rainfall were not recorded.
Location	In and around the Northern Italian city of Pavia
Access & import/export	Permission was granted for access to farms, veterinary clinics and for clinical samples. All appropriate procedures were followed when transporting the isolates.
Disturbance	NA

Reporting for specific materials, systems and methods

We require information from authors about some types of materials, experimental systems and methods used in many studies. Here, indicate whether each material, system or method listed is relevant to your study. If you are not sure if a list item applies to your research, read the appropriate section before selecting a response.

Materials & experimental systems

n/a	Involved in the study	
☒	☐	Antibodies
☒	☐	Eukaryotic cell lines
☒	☐	Palaeontology and archaeology
☒	☐	Animals and other organisms
☐	☒	Human research participants
☒	☐	Clinical data
☒	☐	Dual use research of concern

Methods

n/a	Involved in the study	
☒	☐	ChIP-seq
☒	☐	Flow cytometry
☒	☐	MRI-based neuroimaging

Human research participants

Policy information about studies involving human research participants

Population characteristics	The study was focused on bacterial isolates from stools and rectal swabs from humans (and other non-human biological materials). Full anonymity was provided to the enrolled persons, with only the collection of limited metadata useful for the study (these are available in a linked microreact project and separately in a supplementary table). No additional data from the patients are available to the authors, no follow up was performed.
Recruitment	The samples included stool and rectal swabs from hospital inpatients and outpatients (four different hospitals) and from a nursing home; stool from healthy volunteers.<br>For patients, no selection was performed, and all the samples obtained by the research group through the clinical laboratory of the San Matteo Hospital in Pavia was analyzed, up to the monthly analytical capacity of the research Group.<br>For healthy volunteers, no selection was performed. The study was publicized by word of mouth and through the channels of the Hospital and of the University of Pavia. All the volunteers that contacted us were enrolled. Limited metadata was required (see above) and full anonymity was guaranteed.
Ethics oversight	This study was approved by the Ethical Committee of the San Matteo Hospital in Pavia under number 20170001787 in date 25/05/2017. The proceeding number is 2017000759 and the internal code of the project is 0890170117. The ethical procedure includes written informed consent from all the patients participating in the study.

Note that full information on the approval of the study protocol must also be provided in the manuscript.
